# Evaluation of vicinity-based hidden Markov models for genotype imputation

**DOI:** 10.1186/s12859-022-04896-4

**Published:** 2022-08-29

**Authors:** Su Wang, Miran Kim, Xiaoqian Jiang, Arif Ozgun Harmanci

**Affiliations:** 1 Center for Precision Health, School of Biomedical Informatics, University of Texas Health Science Center, Houston, TX 77030 USA; 2 Department of Mathematics, Hanyang University, Seoul, 04763 Republic of Korea; 3 Center for Secure Artificial Intelligence For hEalthcare (SAFE), School of Biomedical Informatics, University of Texas Health Science Center, Houston, TX 77030 USA

**Keywords:** Genotype imputation, Hidden Markov models, Forward–Backward algorithm, Viterbi algorithm

## Abstract

**Background:**

The decreasing cost of DNA sequencing has led to a great increase in our knowledge about genetic variation. While population-scale projects bring important insight into genotype–phenotype relationships, the cost of performing whole-genome sequencing on large samples is still prohibitive. In-silico genotype imputation coupled with genotyping-by-arrays is a cost-effective and accurate alternative for genotyping of common and uncommon variants. Imputation methods compare the genotypes of the typed variants with the large population-specific reference panels and estimate the genotypes of untyped variants by making use of the linkage disequilibrium patterns. Most accurate imputation methods are based on the Li–Stephens hidden Markov model, HMM, that treats the sequence of each chromosome as a mosaic of the haplotypes from the reference panel.

**Results:**

Here we assess the accuracy of vicinity-based HMMs, where each untyped variant is imputed using the typed variants in a small window around itself (as small as 1 centimorgan). Locality-based imputation is used recently by machine learning-based genotype imputation approaches. We assess how the parameters of the vicinity-based HMMs impact the imputation accuracy in a comprehensive set of benchmarks and show that vicinity-based HMMs can accurately impute common and uncommon variants.

**Conclusions:**

Our results indicate that locality-based imputation models can be effectively used for genotype imputation. The parameter settings that we identified can be used in future methods and vicinity-based HMMs can be used for re-structuring and parallelizing new imputation methods. The source code for the vicinity-based HMM implementations is publicly available at https://github.com/harmancilab/LoHaMMer.

**Supplementary Information:**

The online version contains supplementary material available at 10.1186/s12859-022-04896-4.

## Background

As the cost of DNA sequencing is decreasing, the number of available genome sequences is increasing at a fast pace [1–4]. DNA sequencing is also the fundamental step for technologies such as RNA sequencing and ChIP-Sequencing [5]. Currently, there are millions of genomic sequences available and many more are expected [6–8]. As the genomic data is used more prevalently in the clinic and in translational research [9, 10], the genetic data size is available in many different scenarios, even including the citizen scientists from the general population [11]. Genetic data is deposited widespread in many places (including personal computers and even phones) and it made its way well into the fields of recreational genetics [12]. This is made possible by extensive mapping of the genetic differences between populations and efficient methods that can sift through massive databases for searching for relatives [13]. These are made possible by population-scale projects such as UKBiobank [14].

One of the main uses of genetic data is performing genotype–phenotype associations using genome-wide association studies (GWAS or GWA study) [15–18]. For this, a large cohort is generated and the individuals are genotyped by sequencing. Next, the phenotype of interest (Intelligence quotient, height, body-mass index, blood glucose levels, etc.) is measured from all the individuals. Finally, the measured genotypes for all the variants are tested for association with the GWA studies, most variants are found to be in intergenic regions out of the protein-coding exons. Thus, it is necessary to perform genotyping of the whole genome using, for example, whole-genome sequencing (WGS) to ensure that the causal variants can be accurately detected. Causal variants are the variants that impact a coding or non-coding element (e.g. enhancers) and mechanistically affect the manifestation of a disease or cause a significant phenotypic difference between cases and controls. GWA studies usually detect a variant that is in linkage disequilibrium (LD) [19, 20] and the real causal variant that is most likely associated with the trait must be dissected by a fine-mapping procedure. This, however, is not cost-effective because large samples must be whole-genome sequenced [21]. To get around this, genotyping arrays are used for genotyping and decreasing the cost [22]. The genotyping arrays are designed to genotype only a sparse set of variants from the genome. These variants are then input to in-silico genotype imputation algorithms [23, 24], which impute and “fill-in” the un-genotyped (or untyped for short) variants. The main idea behind the imputation algorithms is to make use of the known haplotype structure of the whole genome and estimate the genotypes of the untyped variants using the genotypes of typed- variants that are correlated at the haplotype level [25]. The haplotype structure arises because the alleles are inherited between generations by a limited number of crossing-overs at the recombination hotspots between homologous chromosomes [26]. This causes long chunks of haplotypes to be inherited as a single unit between parents and children. Although the length of conserved chunks (identity-by-descent segments [27]) decreases as the relationship distance increases, it can still be detected even with 20–25 generations of separation between individuals [28, 29]. The imputation algorithms focus on making use of conserved chunks of haplotypes (i.e., frequent haplotypes) that are shared among unrelated individuals in the population. Imputation methods are also used for imputing variants identified by the RNA sequencing and whole-exome sequencing and for fine mapping of the variants from association studies.

The current state-of-the-art imputation methods such as BEAGLE, Minimac, and IMPUTE suite make use of the hidden Markov model (HMM) [30–34] based approach that is developed by Li and Stephens [35–40]. HMM treats each haplotype as a “state” and analyzes the probabilities of all the “paths” that pass through the states to generate the alleles that are typed by the array [36]. This way, HMM-based methods can assign probabilities to the imputed genotypes using the probabilistic model imposed by the Li–Stephens haplotype model. The HMM takes the typed variants and the reference panel as input and imputes all the variants that exist on the reference panel but are untyped by the genotyping array. While HMM models provide good accuracy of imputation, they may fail at imputation of rare variants as these variants are represented on at least as rare haplotypes [41]. However, as the size of reference panels increases, the rare variants can be more accurately predicted [42].

Here, we focus on Li–Stephens HMM-based imputation models and assess the performance of “vicinity-based HMMs”, i.e., the HMM evaluates the paths over only a short stretch of variants around the untyped variants. While several methods have tested different parametrizations of the state-of-the-art methods, we implemented the vicinity-based HMM methods to have full control over how the parameters impact the imputation. Evaluation of the imputation parameters is justifiable since it has been previously shown that genome has varying “imputability” [43], i.e., some variants are more easily imputed while others are less imputable because of complex haplotype structures around them. Thus, it is useful to study the parameters of the vicinity-based models in detail for tuning the parameters of new resource intensive methods such as Deep Learning-based and secure imputation methods. In this study, we focus on the impact of different parameters related to vicinity-based imputation models and how they impact the accuracy of imputation. While we do not explicitly aim for generating the optimal vicinity-based imputation parameters for different regions in the genome, we provide evidence that the vicinity-based models with a fairly constrained set of parameters can provide good imputation accuracy even without a more extensive optimization over the genome. These parameters can serve as a starting point while searching for the vicinity-specific imputation parameters that are optimal with respect to accuracy or other application specific considerations, e.g. security-vs-performance.

The locality-based approaches have been used in different scenarios, for instance with linear imputation models and with Deep Learning-based imputation models [44–46], where the imputation is performed on the typed variants that are in the vicinity of the untyped variant. Also, IMPUTE and BEAGLE make use of a sliding-window, as long as 40 centimorgans (cM) to cut corners in computation. This parameter was not extensively assessed in terms of its impact on imputation accuracy, especially for much shorter window lengths. We implemented the per-position posterior probability estimation (we refer to this as the “forward–backward” or “FB” method) by the forward–backward algorithm. We also implement the inference of the maximum-likelihood HMM path (referred to as the Viterbi method), which represents the most likely mosaic of reference haplotypes that gives rise to the genotypes of the typed variants. On these methods, we analyze the size of the window, positioning of the target within the window, and the number of typed variants on the window. We also report effect of population-specific imputation by analyzing different reference populations used in imputation. It should be noted that we focus on the phased genotype imputation problem, i.e., we assume that the genotypes are phased. This is a reasonable assumption since pre-phasing was shown to improve the time complexity of the imputation method substantially while conferring a very small performance penalty [39].

One of the main advantages of the locality-based approaches is that they can be constrained in terms of computational requirements without the need to load the whole genome into memory or running the HMM inference methods on whole chromosomes or very long stretches. This way, the architecture of imputation algorithms on a cloud can be structured accordingly, for example, by using different models in different parts of the genome. On another front, the recent efforts to develop privacy-aware genotype imputation methods make use of the vicinity-based models to perform imputation while genotype data confidentiality is preserved [47, 48]. These methods can potentially serve as alternative for genotype imputation servers such as Michigan Imputation Server [49], which provide imputation-as-a-service. In these servers, the genotype data is processed in plain form and anyone can access the genotype data, which makes it fairly concerning to make use of these services when data is from vulnerable or underrepresented populations. As the genetic privacy is becoming an important topic of public discussion, it is necessary to develop more efficient imputation methods that can be used to build the imputation services with privacy-by-design principles. While there have been attempts (including our group) to build secure imputation tools [47, 48, 50, 51] using trusted execution methods and homomorphic encryption techniques [52], these methods are bound by computational requirements [53]. Therefore, our study can inform these methods about the locality parameters that must be considered and evaluated for decreasing computational requirements and maintaining the imputation accuracy while providing privacy and confidentiality for the genetic data. The implementation of the vicinity-based HMMs, named LoHaMMer, is publicly available to download from https://github.com/harmancilab/LoHaMMer.

## Results

We briefly describe the HMM-based imputation techniques, the parameters, and the evaluation approach. We finally present the imputation accuracy evaluations.

### Overview of the vicinity-based HMMs

**Fig. 1:**
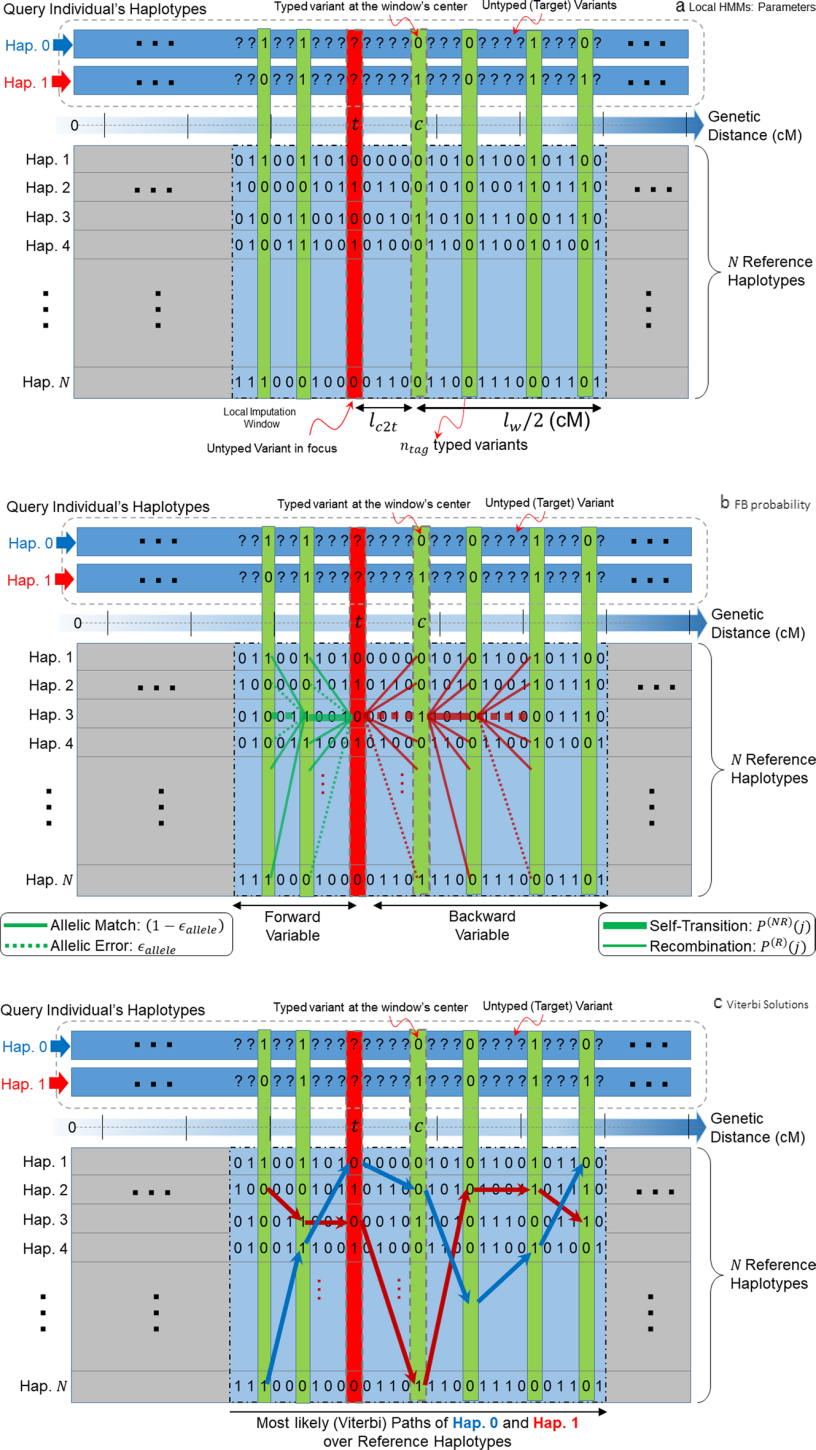
**a** Illustration of the local imputation setup. Query individual’s parental haplotype copies (“Hap. 0” and “Hap. 1”) are shown in two rectangles in top, which are strings of {0,1}. 0 and 1 indicate a reference and alternate allele, respectively, for corresponding variants. The untyped variants are indicated with “?” to indicate their alleles are not known. The genetic distance (in centimorgans) are shown with the blue arrow and is used to track the center position (indicated with ‘*c*’) of the window and the target untyped variant in focus (indicated with “*t*”). The reference haplotypes are shown in the box below wherein each row corresponds to a haplotype. Given the local window of radius ${l}_{w}$ the window is illustrated in the dashed rectangle whose center is shown at the genetic position $c$ and for the target variant at position $t$. The typed variants are shown in green rectangles and the untyped target is shown in the red rectangle, whose alleles on the query haplotypes are shown with question marks. **b** Illustration of the forward and backward variables for the emission of the allele sequence on “Haplotype 0”. For the 3rd haplotype at the untyped variant, the incoming paths (forward variable) are illustrated with green lines. Each green line stems from a haplotype (i.e., state) indicating the emission of one of the alleles on the corresponding haplotype. The dashed paths indicate an allelic mismatch between individual’s haplotype (Hap. 0) and the reference haplotype. These paths are penalized with allelic error probability (${\epsilon }_{allele}$) at the forward and backward variables. The incoming paths for variant positions further to the left are also shown, which depict the exponential increase in the number of paths that are evaluated in the hidden Markov model. The outgoing transitions on the right side of the target variant are shown with red lines. The self-transitions are shown with heavier lines compared to the non-self-transitions to depict that they have higher probabilities of occurrence, i.e., the probability of maintaining a haplotype is higher than creating a recombination event. **c** Two Viterbi paths are shown with the transitions along the haplotypes corresponding to the haplotypes of query individual, “Hap. 0” and “Hap. 1”

Genotype imputation is summarized in Fig. 1. The genotype imputation process takes as input the variant phased genotypes matrix, ${G}_{M\times V}$, individuals. As we are evaluating the phased imputation process, $G$ is pre-phased using a phasing algorithm such as Eagle [49] (Fig. 1a). ${G}_{i,j}$ holds the phased genotype of the ${j}^{\mathrm{th}}$ variant for the ${i}^{\mathrm{th}}$ individual, i.e., ${G}_{i,j}^{(h)}\in \left\{\mathrm{0,1}, \mathrm{\varnothing }\right\}, 1\le i\le M, 1\le j\le V$, where $h$ indicates the paternal/maternal copy for the genotype, i.e. $h\in \{\mathrm{0,1}\}$. ${G}_{i,j}^{(h)}=\mathrm{\varnothing }$ indicates the missing genotype that will be imputed using the reference panel. We denote the set of indices of the untyped variants with ${j}_{\varnothing }=\left\{j | {G}_{i,j}^{(h)}=\varnothing \right\}$. Imputation also takes the reference genotype matrix containing ${H}_{N\times V}$ of $N$ haplotypes over the same $V$ variants that correspond to the columns of $G$. Similar to $G$, ${H}_{i,j}\in \left\{\mathrm{0,1}\right\},1\le i\le N, 1\le j\le V$.

#### Li–Stephens Markov model

Our evaluations use the Markov model defined by the standard Li–Stephens model [35], where the haplotypes of each query individual are modeled as a “mosaic of the reference haplotypes” such that pieces of reference haplotypes (consecutive variant alleles on a haplotype) are concatenated to each other. This model describes a probability distribution on possible “paths” that pass over the reference haplotypes (Fig. 1b, c). In this model, the transitions between the haplotypes and errors on the haplotypes are probabilistic. In the simplest sense, the minimal number of haplotype transitions and allelic errors can be thought of as the most likely path that describes the query haplotype. The basic idea is to pin-down the typed variant alleles on the paths, and estimate the marginal probabilities of alleles at the untyped variants:

1 $$\forall j\in {j}_{\varnothing }; P\left({G}_{i,j}^{\left(h\right)}=0\right)={\sum }_{k}\left(\begin{array}{c}Probability of {k}^{th} path such \\ that {j}^{th}variant allele is 0\end{array}\right)$$

In this model, the haplotypes of the reference panel correspond to the states of the Li–Stephens Markov model. Each state (haplotype) emits an allele at a variant position $1\le j\le V$. In addition, the transitions between the states (i.e., the switches between haplotypes) at variant $j$ are dependent only on the genetic distance between the variants at indices $j$ and $\left(j+1\right)$. The genetic distance measures the probability of recombination taking place between these two variants. In the Markov model, recombination corresponds to a state-switch whereby the state (i.e., the haplotype) makes a transition to a new state. However, the recombinations occur as homologous chromosomes crossover in the course of meiosis. The rate of recombinations changes depending on the position on the genome, i.e., some parts of the genome are more likely to harbor recombinations than others. Thus, the prevalence of recombination events along the genome is quantified in terms of genetic distance that is measured in centimorgans (cM), a measure of recombination probability between two loci. Given two variants at indices $\left(j-1\right)$ and $j$, the probability of recombination is modeled as:

2 $${P}_{j}^{\left(R\right)}=\frac{1}{N}\times \left(1-\mathrm{exp}\left(-4\times {N}_{e}\times \frac{\Delta {R}_{j}}{N}\right)\right), {\Delta R}_{j}={R}_{j}-{R}_{j-1}$$

where ${P}_{j}^{\left(R\right)}$ denotes the probability that there is a recombination event (i.e. Markov chain stays on the same state), ${R}_{j}$ denotes the interpolated cumulative genetic distance of variant at index $j$ (See Methods for exact definition), ${\Delta R}_{j}$ denotes the genetic distance between variants at indices $\left(j-1\right)$ and $j$, and ${N}_{e}$ denotes the effective population size. It is important to note that the probability of recombination depends only on the position of the variant and not the actual haplotype. This is widely used in HMM-based imputation methods to decrease computational costs. The probability of that a recombination does not take place can be computed from ${P}_{j}^{\left(R\right)}$:

3 $${P}_{j}^{\left(NR\right)}=1-\left(\left(N-1\right)\times {P}_{j}^{(R)}\right)$$

where all recombination events are accounted for and removed from 1 and ${P}_{j}^{\left(NR\right)}$ indicates that there is no recombination between variants at indices $(j-1)$ and $j$. From the above equation for ${P}_{j}^{\left(R\right)}$, Increasing population size implies a higher probability of recombination, i.e., larger effective population size indicates more complex recombination patterns as the probability of switching between haplotypes (or states) increases. Given the query individual’s phased genotypes, ${G}_{i,j}^{\left(h\right)}$ and the reference haplotype data, ${H}_{a,j}$, HMM is defined based on these equations using the transition and emission probabilities. The transition and emission probabilities are formulated as

4 $${\tau }_{j}\left(b\to a\right)=\left\{\begin{array}{c}{P}_{j}^{\left(NR\right)} ;a=b\\ {P}_{j}^{\left(R\right)} ;a\ne b\end{array}\right.$$

5 $${e}_{j}\left({G}_{i,j}^{\left(h\right)},a\right)=\left\{\begin{array}{c}{{\epsilon }_{allele}=10}^{-4}; {H}_{a,j}\ne {G}_{i,j}^{\left(h\right)}\\ 1-{\epsilon }_{allele} ; {H}_{a,j}={G}_{i,j}^{\left(h\right)}\end{array}\right.$$

where ${\tau }_{j}\left(b\to a\right)$ denotes the transition probability from haplotype $b$ to $a$ at variant index $j$ from the previous variant at index $\left(j-1\right)$ and ${e}_{j}\left({G}_{i,j}^{\left(h\right)},a\right)$ denotes the emission probability of the allele ${G}_{i,j}^{\left(h\right)}$ from the ${a}^{\mathrm{th}}$ haplotype. The emission probability depends on the alleles of the query individual; if the allele on the ${a}^{\mathrm{th}}$ haplotype matches query individual’s allele, a high emission probability is assigned, otherwise allele error probability, ${\epsilon }_{allele}$, is assigned as the emission probability.

Using the above equations and Li–Stephens Model, we use two approaches for inferring the haplotype states at every typed variant.

#### Inference of marginal state (haplotype) probabilities

First approach the estimation of per-typed-variant estimate of posterior probabilities of each haplotype and assignment of the forward–backward marginalization-based estimate of the alleles at the untyped variants (Fig. 1b). For this, we make use of the forward–backward algorithm [54], which is a well-known dynamic programming algorithm that is used to efficiently compute the state probabilities at each step of the HMM as

6 $$P\left( {S_{{i,j}}^{{\left( h \right)}} = a,~G_{{i,~\left[ {1,V} \right]}}^{{\left( h \right)}}} \right) = \underbrace {{P\left( {S_{{i,j}}^{{\left( h \right)}} = a,~G_{{i,\left[ {1,j} \right]}}^{{\left( h \right)}} } \right)}}_{{Forward~Variable}} \times \underbrace {{P\left( {G_{{i,\left[ {j + 1,V} \right]}}^{{\left( h \right)}}|S_{{i,j}}^{{\left( h \right)}} = a} \right)}}_{{Backward~Variable}}$$

where ${S}_{i,j}^{\left(h\right)}$ denotes the state (haplotype) of the HMM at variable index $j$ for individual $i$’s parental copy $h$ ($h\in \{\mathrm{0,1}\}$), ${G}_{i,[1,j]}^{(h)}$ denotes the sequence of alleles for variants in $[1,j]$ on individual $i$’s parental copy $h$, and $P\left({S}_{i,j}^{(h)}=a, {G}_{i,[1,j]}^{(h)}\right)$ is the forward-variable and it denotes the probability of emitting the allele sequence ${G}_{i,[1,j]}^{(h)}$ given that HMM is at state $a$ at the variant position $j$. Backward-variable is similarly defined for the rest of the allele sequence that is backward of *j*th variant, i.e., ${G}_{i,[j+1,V]}^{\left(h\right)}$. The forward and backward variables are computed using efficient recursion relations (See Methods) [55, 56]. The relation in (6) follows from conditional independence of ${G}_{i,[j+1,V]}^{\left(h\right)}$ and ${G}_{i,[j+1,V]}^{\left(h\right)}$, given ${S}_{i,j}^{(h)}=a$. After the forward and backward variables at each variant position $j$ and for each state $a$ are computed, we can estimate the posterior probability of each allele at each untyped position:

7 $$\forall j\in {j}_{\varnothing }, P\left({G}_{i,j}^{\left(h\right)}=t\right)={\sum }_{a\le N}P\left({S}_{i,j}^{\left(h\right)}=a , {H}_{a,j }=t\right) , t\in \left\{\mathrm{0,1}\right\}$$

The untyped variant allele $t$’s probability is estimated by marginalizing over the states $a$ for which the corresponding haplotype has an allele $t$. As we describe below, we evaluate 2 different approaches for marginalizing over the haplotypes.

#### Maximum-likelihood mosaic-haplotype (Viterbi)

While forward–backward algorithm focuses on marginalizing at a specific variant, Viterbi algorithm aims to predict the most likely “path” along the haplotypes (Fig. 1c) so that one single haplotype is a “mosaic” of the reference haplotypes. Conceptually, the forward–backward algorithm calculates the marginal probability of each haplotype at each variant while Viterbi analyzes the overall probability of all variants to identify the optimal mosaic haplotype. To compute the most likely haplotype, the overall probability of the state sequence conditioned on the haplotype’s allele sequence is maximized. We denote this as

8 $${\overset{\lower0.5em\hbox{$\smash{\scriptscriptstyle\smile}$}}{S}}_{i,\left[1,V\right]}^{\left(h\right)}=\underset{{S}_{[1,V]}}{\mathrm{argmax}}\left\{P\left({S}_{[1,V]} , {G}_{i,[1,V]}^{\left(h\right)}\right)\right\}$$

where $$P\left({S}_{[1,V]} , {G}_{i,[1,V]}^{\left(h\right)}\right)$$ denotes joint probability of the state sequence $${S}_{[1,V]}$$ and the corresponding allele sequence of all variants in $$\left[1,V\right]$$ for *i*th individual that is emitted by the state sequence. $${\overset{\lower0.5em\hbox{$\smash{\scriptscriptstyle\smile}$}}{S}}_{i,[1,V]}^{(h)}$$ denotes the state sequence that maximizes the probability for *i*th individual’s haplotype $$h$$ (Fig. 1c). This state sequence represents the most likely mosaic haplotype that gives rise to the variant alleles$${G}_{i,[1,V]}^{\left(h\right)}=({G}_{i,1}^{(h)},{G}_{i,2}^{(h)}, \ldots ,{G}_{i,V}^{(h)})$$. The state sequence can be inferred using a dynamic programming algorithm, namely the Viterbi algorithm [57] that efficiently identifies the maximum-likelihood state sequence similar to the forward algorithm.

After the most likely state sequence is computed using the Viterbi algorithm, we assign the alleles for untyped variants using the alleles that are on this state sequence:

9 $$\forall j\in {j}_{\varnothing }, {\overset{\lower0.5em\hbox{$\smash{\scriptscriptstyle\smile}$}}{G}}_{i,j}^{\left(h\right)}={H}_{{\overset{\lower0.5em\hbox{$\smash{\scriptscriptstyle\smile}$}}{S}}_{i,j}^{\left(h\right)},j}$$

Here, $${\overset{\lower0.5em\hbox{$\smash{\scriptscriptstyle\smile}$}}{G}}_{i,j}^{\left(h\right)}$$ denotes the allele on the most likely haplotype for the untyped variant $$j$$ that is assigned to individual $$i$$’s haplotype $$h$$. The Viterbi algorithm does not immediately assign a score for each imputed allele. We aggregate the vicinity information to assign a score for the imputed allele.

#### Locality parameters of imputation

We evaluate the effect of changing parameters on the accuracy of genotype imputation. The forward–backward and Viterbi-based imputation algorithms sequentially analyze the variants while keeping track of the scores and probabilities for each state. They can be performed using all of the variants on each chromosome as the LD information is confined generally to individual chromosomes and inter-chromosomal LD information, while detectable, are very weak [58]. These are out-of-scope of the imputation methods that we evaluate. Using whole chromosomes in imputation enables the algorithm to integrate the linkage information from all positions on the chromosomes. On the other hand, the linkage information tends to decrease quickly while imputing an untyped variant, e.g., the identity-by-descent segment length (length of conserved haplotypes) decreases quickly among generations (25 generations separation have on average 2 cM conservation [28]). This information can be integrated into forward–backward (Fig. 1b) and Viterbi-based (Fig. 1c) imputation by a sliding-window where the variants outside a local window are not used for imputation. This can help decrease the computational requirements. For example, BEAGLE uses a large sliding window (length 30 cMs) and merges the consecutive windows to infer the forward and backward variables. In our study, we run forward–backward and Viterbi algorithms solely on the local windows around the untyped variants and use these “vicinity-based HMMs” to impute the untyped variants. For instance, if we are using a local window of length 0.5 centimorgan (cM), the most likely state sequence is computed only for the local vicinity of the is assigned using

10 $${\overset{\lower0.5em\hbox{$\smash{\scriptscriptstyle\smile}$}}{S}}_{i,\left[k,l\right]}^{\left(h\right)}=\underset{{S}_{[k,l]}}{\mathrm{argmax}}\left\{P\left({S}_{[k,l]} , {G}_{i,[k,l]}^{\left(h\right)}\right)\right\}, \left|{R}_{k}-{R}_{l}\right|\approx 0.5 \mathrm{cM}$$

where $${R}_{k}$$ denotes the interpolated cumulative genetic distance of variant $$k$$ (See Methods). In (10), the state sequence, $${\overset{\lower0.5em\hbox{$\smash{\scriptscriptstyle\smile}$}}{S}}_{i,\left[k,l\right]}^{\left(h\right)}$$, is confined to the variant indices $$[k,l]$$ whose genetic distance is approximately 0.5 cMs. The forward–backward computations are similarly confined to the local windows based on genetic distance cutoffs.

We test different local window lengths and evaluate the impact of window length on the allele imputation accuracy. We utilize a sliding window with lengths from 0.1 upto 1 cMs and compute the imputation accuracy (See Metrics). Another important factor is the positioning of the untyped target variant within the local window (Fig. 1a). It is expected that the LD information can be integrated more accurately if the untyped variant is centered around the local window. It is, however, not clear to what extent “target-to-center distance” affects the imputation accuracy. For each untyped target variant, we first identified the typed variants that will be used for imputation that satisfies the local window length and target-to-center distance criteria using Viterbi and forward–backward approaches with the selected population size and allelic probability assignment procedure.

#### Evaluation setup and metrics

We use genotype data from the 1000 genomes Project’s Phase 3 [59]. We focus on the variants on chromosomes 19, 20, and 22 for extensive evaluation and exclude the multi-allelic SNVs and indels. Among these data, chromosome 22 is used to evaluate different parameter combinations. To decrease computational requirements with the parameter combinations, we focused on the region chr22:25,000,000–35,000,000. In the evaluations, we randomly selected 1000 individuals as the phased reference panel and 200 individuals (with known genotypes) for estimating evaluation. After we evaluated the parameters, we selected the optimal parameter set and validated the imputation on chromosomes 19 and 20. To define the typed (tag) variants, we extracted the positions of the variants that are genotyped on the Illumina Duo 1 M genotyping array platform [60] as it was recently used in our previous study [47]. This enables us to perform evaluations on a realistic test case as the Illumina’s array is used in several large-scale projects including the HAPMAP project [60]. We used all the variants that map to the positions that overlap with Illumina Duo platform as the typed variants and the remaining variants are assigned as untyped variants that are imputed. After extracting the variants, we phased the genotypes using EAGLE2 [49]. The phased typed variants are input to LoHaMMer with different parameters for imputation. After the untyped variants are imputed, (1)) Genotype concordance (all and non-reference genotypes), and (2) Precision-Recall curves based on the imputed probabilities. We compare the implemented locality-based HMMs with BEAGLE, which is used as the baseline method for imputation. The imputation accuracy is classified among variants with respect to the range of minor allele frequency (MAF) and with respect to the chromosomal position.

### Evaluation of imputation accuracy with changing locality parameters

**Fig. 2:**
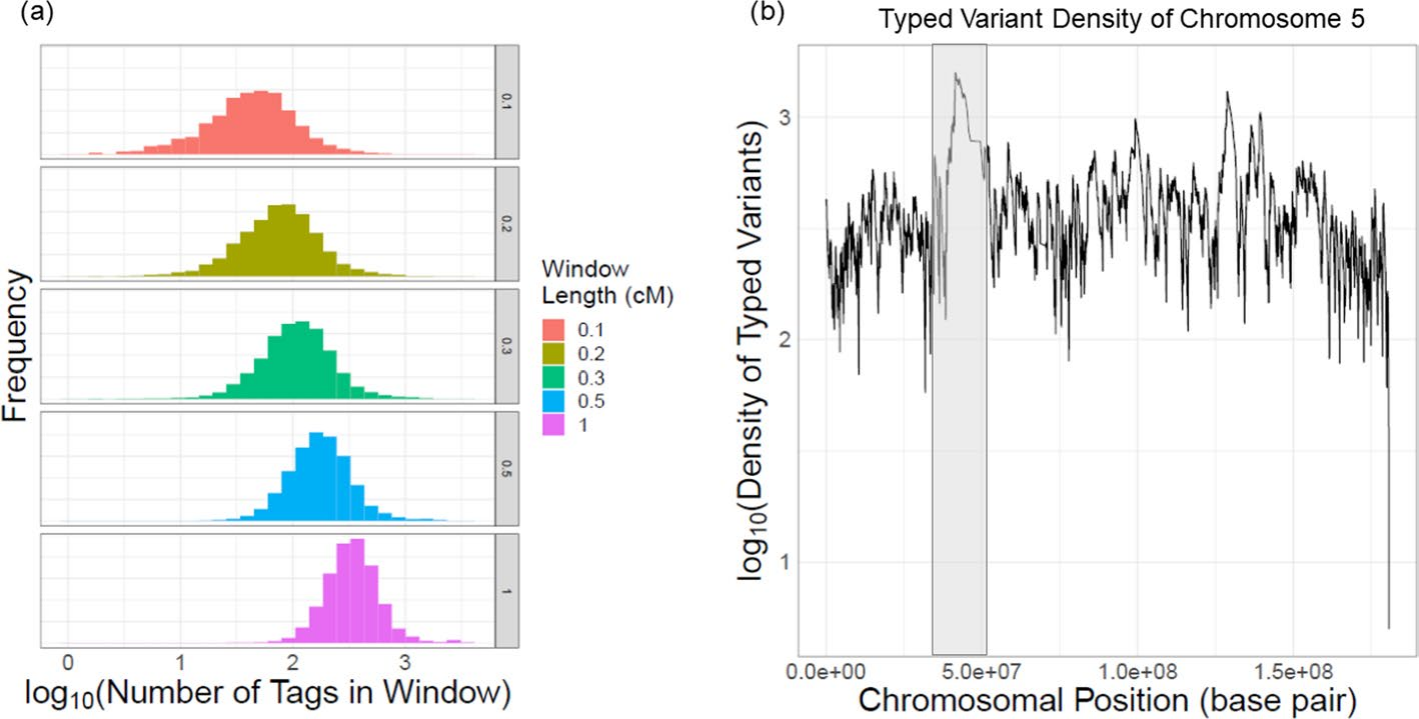
Typed variant statistics. **a** The distribution of typed variant number for different window lengths for common untyped variants. **b** The number of typed variants for 1 cM window length over the chromosome 5 coordinates. X-axis shows the chromosomal position and Y-axis shows the tag variant density. The centromere is indicated with a grey rectangle

For assessing the imputation accuracy with changing parameters, the variants are classified into “common” (MAF > 0.05) and “uncommon” (MAF < 0.05) variants. We tested the impact of the 4 different parameters including window length (${l}_{w}$), target-to-center distance (${l}_{c2t}$), and number of typed variants in the window (${n}_{tag}$). Here, rather than computing all parameter combinations, we selected a range for each parameter and we evaluated the impact of one parameter while keeping others constant. We used $\left({l}_{w},{N}_{e},{l}_{c2t},{n}_{tag}\right)=({\mathrm{0.3,10}}^{4},\mathrm{0.05,10000})$ as the default parameter values. While ${n}_{tag}$ is set to a large value of 10,000, the number of local typed variants depend on the locality window length (${l}_{w}$). Figure 2a shows the distribution of number of typed variants with different window length parameter. For the longest tested window length of 1 cM (Bottom panel in Fig. 2a), we observed that majority of the windows contain less than 1000 typed variants. We also observed there is generally uniform coverage of typed variants along the chromosomes (Fig. 2b, Additional file 1: Fig. S1).

#### Local window length (${\mathbf{l}}_{\mathbf{w}})$

**Fig. 3:**
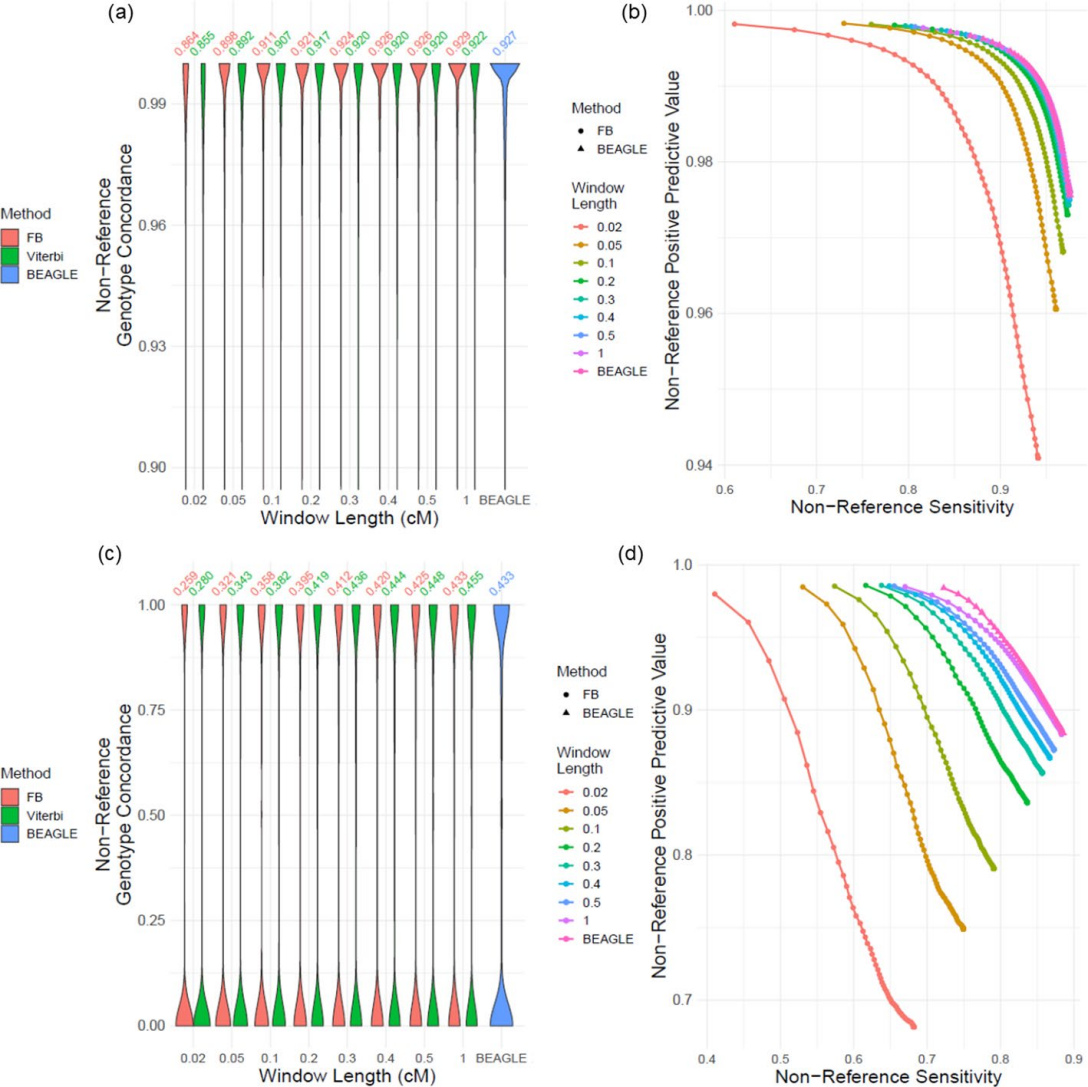
Effect of changing window length on accuracy. **a** Distribution of non-reference genotype concordance for changing window length (${l}_{w}$) for common variants. **b** The PR-curve for non-reference genotypes with respect to changing ${l}_{w}$ for common variants. **c** Distribution of non-reference genotype concordance for uncommon variants with respect to ${l}_{w}$. **d** Non-reference genotype PR-curves for changing ${l}_{w}$ for uncommon variants

We first evaluated the impact of local window length on the imputation accuracy. We used local window lengths of ${l}_{w}\in \left\{0.02, 0.05, 0.1, 0.2, 0.3, 0.4, 0.5, 1\right\}$ cM. Figure 3a, b show the non-reference genotype concordance distribution and the precision-recall curve for non-reference genotypes of common variants for different window lengths. As expected, the accuracy increases with increasing window lengths. For window lengths above 0.3 cMs, we observed that there is around 0.5% increase in the non-reference genotype concordance when forward–backward and BEAGLE are compared (Fig. 3a, Additional file 1: Fig. S2a). For the uncommon variants, the window lengths greater than 0.5 cMs exhibit very similar behavior as BEAGLE with 0.8% difference (Fig. 3c). The precision-recall curves for uncommon variants show that the curves are very close to each other above 0.3 cMs for non-reference genotypes (Fig. 3d). These results indicate that ${l}_{w}>0.3$ cM is the minimum window length with comparable accuracy as BEAGLE. For uncommon variants, we observed that concordance are at the high or low accuracy regimes (Fig. 3c) for both BEAGLE and LoHaMMer. The PR curves for all genotypes of uncommon variants demonstrate a fairly steady pattern of increase in the accuracy (Additional file 1: Fig. S2b) with fairly similar accuracy for ${l}_{w}>0.3$.

#### Target-center distance (${\mathbf{l}}_{\mathbf{c}2\mathbf{t}}$)

**Fig. 4:**
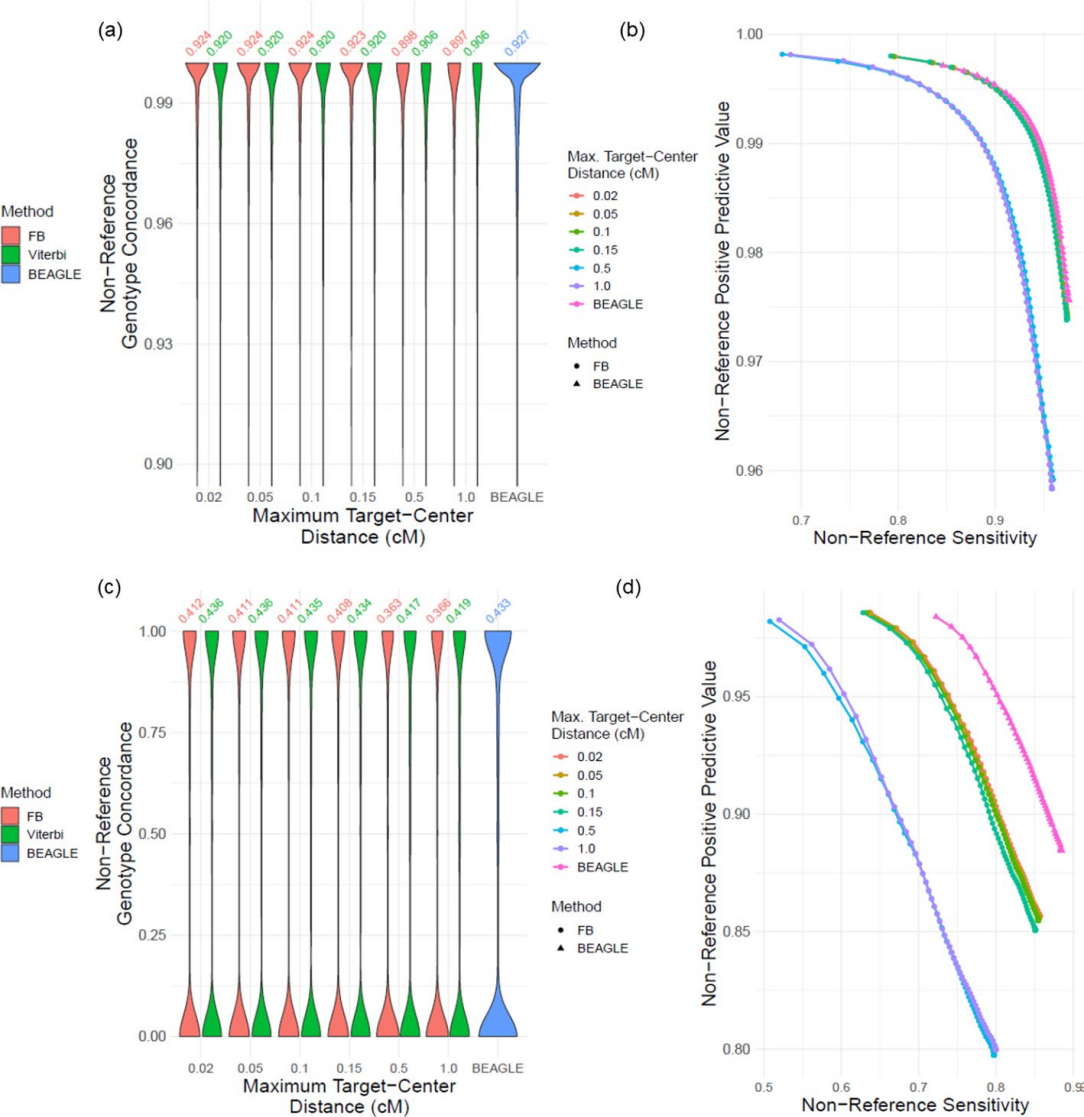
Effect of changing target-to-center distance on accuracy. **a** Distribution of non-reference genotype concordance for changing target-center distance (${l}_{c2t}$) for common variants. **b** The PR-curve for non-reference genotypes with respect to changing ${l}_{c2t}$ for common variants. **c** Distribution of non-reference genotype concordance for uncommon variants with respect to changing ${l}_{c2t}$. **d** Non-reference genotype PR-curves for changing ${l}_{c2t}$ for uncommon variants

The positioning of the target variant, ${l}_{\mathrm{c}2\mathrm{t}}$ (target-center distance), in the imputation window is another parameter that can impact imputation accuracy (Fig. 1a). We tested the imputation of accuracy with increasing target-center distance values, ${l}_{\mathrm{c}2\mathrm{t}}\in \{0.02, 0.05, 0.1, 0.15\}$ cM. We use genetic distance as the measurement of unit for these parameters since it is the most natural choice (Methods). Non-reference genotype concordance for common variants is shown for different center-target distance values indicating a visible impact of target-center distance (Fig. 4a, b). We observed that the imputation accuracy decreases as ${l}_{\mathrm{c}2\mathrm{t}}$ increases. This indicates that the haplotype and LD information from the two sides of the untyped variant should be balanced. For ${l}_{\mathrm{c}2\mathrm{t}}<0.15$ cM, we observed that the local window-based imputation provides comparable accuracy. For uncommon variants, we observed a similar pattern in the terms of non-reference genotype PR curves (Fig. 4c, d). Similar results are found for non-reference concordance and all genotype PR curves (Additional file 1: Fig. S3a) and for uncommon variants (Additional file 1: Fig. S3b).

#### Maximum number of typed variants in window (${\mathbf{n}}_{\mathbf{t}\mathbf{a}\mathbf{g}}$)

**Fig. 5:**
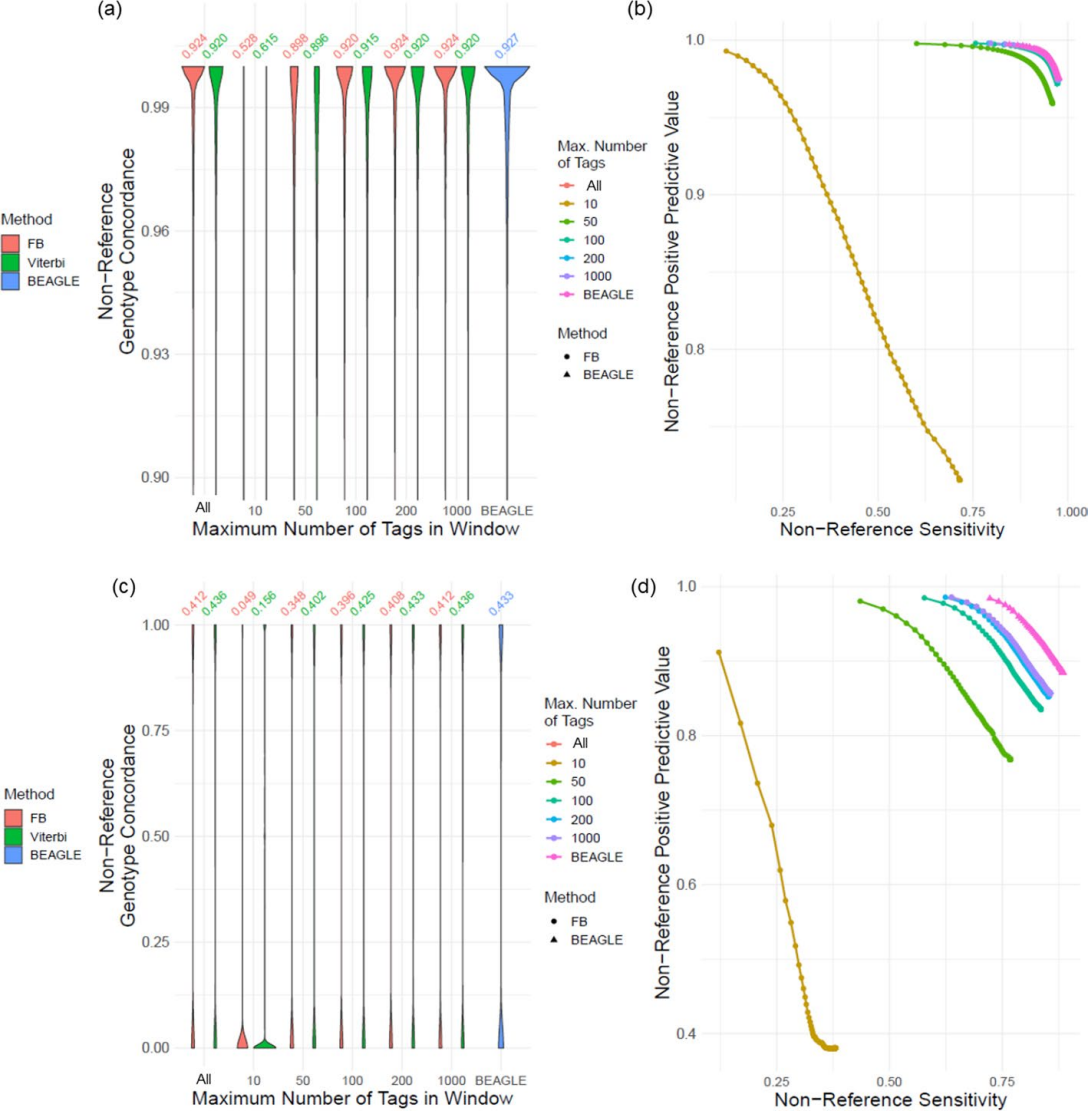
Effect of changing maximum typed variant numbers, ${\mathrm{n}}_{\mathrm{tag}}$. **a** Distribution of non-reference genotype concordance for changing ${n}_{tag}$, for common variants. ‘All’ indicates that all typed variants were used in imputation. **b** The PR-curve for non-reference genotypes with respect to changing ${n}_{tag}$ for common variants. **c** Distribution of non-reference genotype concordance for rare variants with respect to changing ${n}_{tag}$. **d** Non-reference genotype PR-curves for changing ${n}_{tag}$ for uncommon variants

The next set of parameters we tested are the number of typed variants that are used for imputation. For this, we subsampled the typed variants in each window such that the number of typed variants is bounded by the maximum number of typed variants. For this, we evaluated the impact of changing ${n}_{tag}\in \{10, 50, 100, 200, 1000$}. The typed variants in the windows that harbor less than ${n}_{tag}$ typed variants are used as they are. Figure 5a, b show non-reference genotype concordance and PR curve, respectively. For ${n}_{tag}$ greater than 100 variants, we observed that the accuracy levels out with a slight increase, for common variants (Additional file 1: Fig. S4a). For uncommon variants, we observed that the genotype accuracy flattens out around ${n}_{tag}=200$, and PR curves exhibit similar patterns although the imputation accuracy is fairly low for all parameter selections (Fig. 5c, d, Additional file 1: S4b). For uncommon variants, using all of the typed variants in the windows is more suitable as this parameter impact accuracy strongly.

### Ancestral mismatches between reference and query samples

**Fig. 6:**
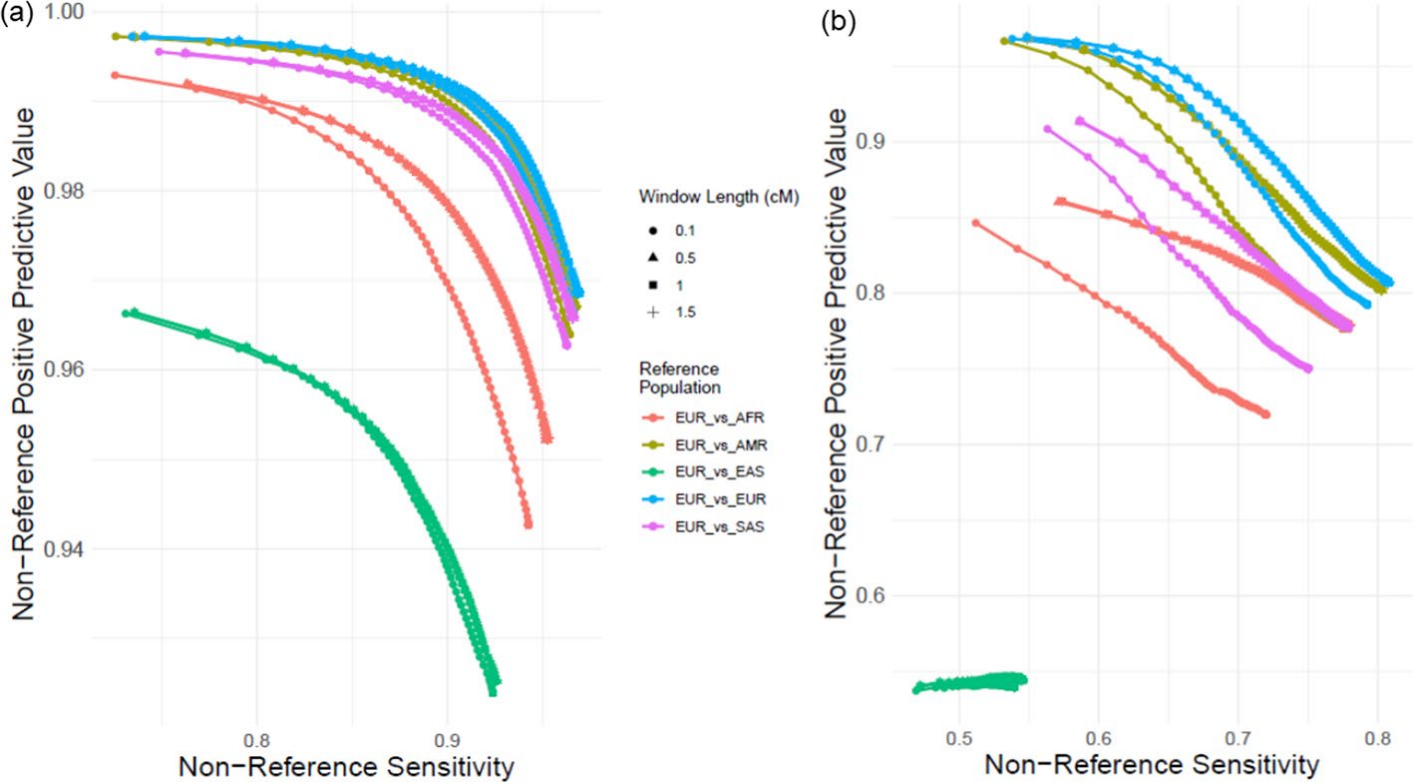
Impact of ancestral mismatches between reference panel and query individual. **a** The non-reference PR curves with changing window lengths for 5 different reference panels for common variants. Different colors correspond to different reference panel. Different dot shapes depict different window lengths. **b** PR curves for uncommon variants with respect to different reference panels (color) and window lengths (shape)

We next tested how the mismatches between the genetic ancestries of query individuals and the individuals in the reference panel affect the accuracy. For this, we used individuals of European descent (Super population EUR) as the query individuals. We used 4 other superpopulations as reference panels: Americas (AMR), African (AFR), East Asian (EAS), South Asian (SAS). As the baseline, we also used European panel as the matching reference panel in imputation. For each of the 5 query-reference panel pairs (including EUR as reference), we performed imputation on the common and uncommon benchmarking variants on chromosome 22 using different window length parameters ${l}_{w}=\left\{0.1, \mathrm{0.5,1},1.5\right\}$. Figure 6 shows the non-reference concordance for different reference populations and window lengths. As expected, the accuracy is highest for the matching EUR reference population followed by AMR population, which is known to contain large amount of EUR admixture [61]. EAS reference panel exhibits the lowest imputation accuracy. While the increasing window length increases accuracy for all reference populations, we observed that highest improvement is attained for AFR reference population. This can potentially be underpinned by recent admixtures of the individuals from European and African descent [62]. In summary, our results show that the parameters may need to be re-parametrized when population-specific reference panels are used.

### Time and memory requirements

**Fig. 7:**
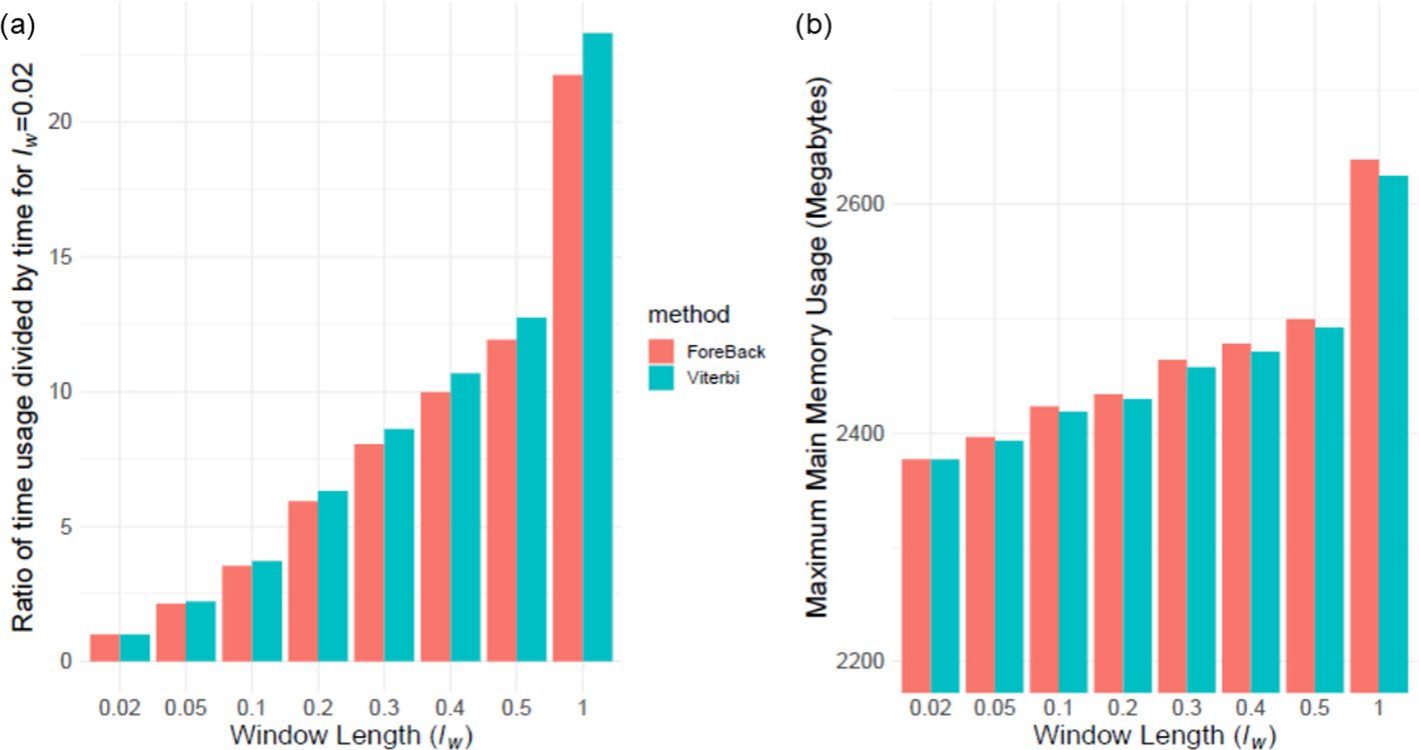
Time (**a**) and memory usage (**b**) of forward–backward (FB) and Viterbi methods on benchmarking dataset for different window lengths shown in x-axis

We tested how the time and memory requirements of vicinity-based HMMs with respect to the increasing window length parameter. We measured the time and memory usage of forward–backward and Viterbi methods using window lengths of $\{0.02, 0.05, 0.1, 0.2, 0.3, 0.4 0.5, 1.0\}$ centimorgans. The time (Fig. 7a) and memory usage (Fig. 7b) of both methods increase linearly with respect to window length. In general, Viterbi requires more time than forward–backward method. This stems from the fact that Viterbi method utilizes many inefficient branching operations that are necessary to identify the optimal paths in Viterbi recursions in (24). We would like to emphasize that our current implementation is optimized for ease of benchmarking. For example, we compute forward and backward variables from scratch for each window and this is not necessary since large number of windows overlap and the variables can be re-used. We discuss numerous approaches for optimizations in Discussion Section.

### Accuracy on chromosomes 19 and 20

**Fig. 8:**
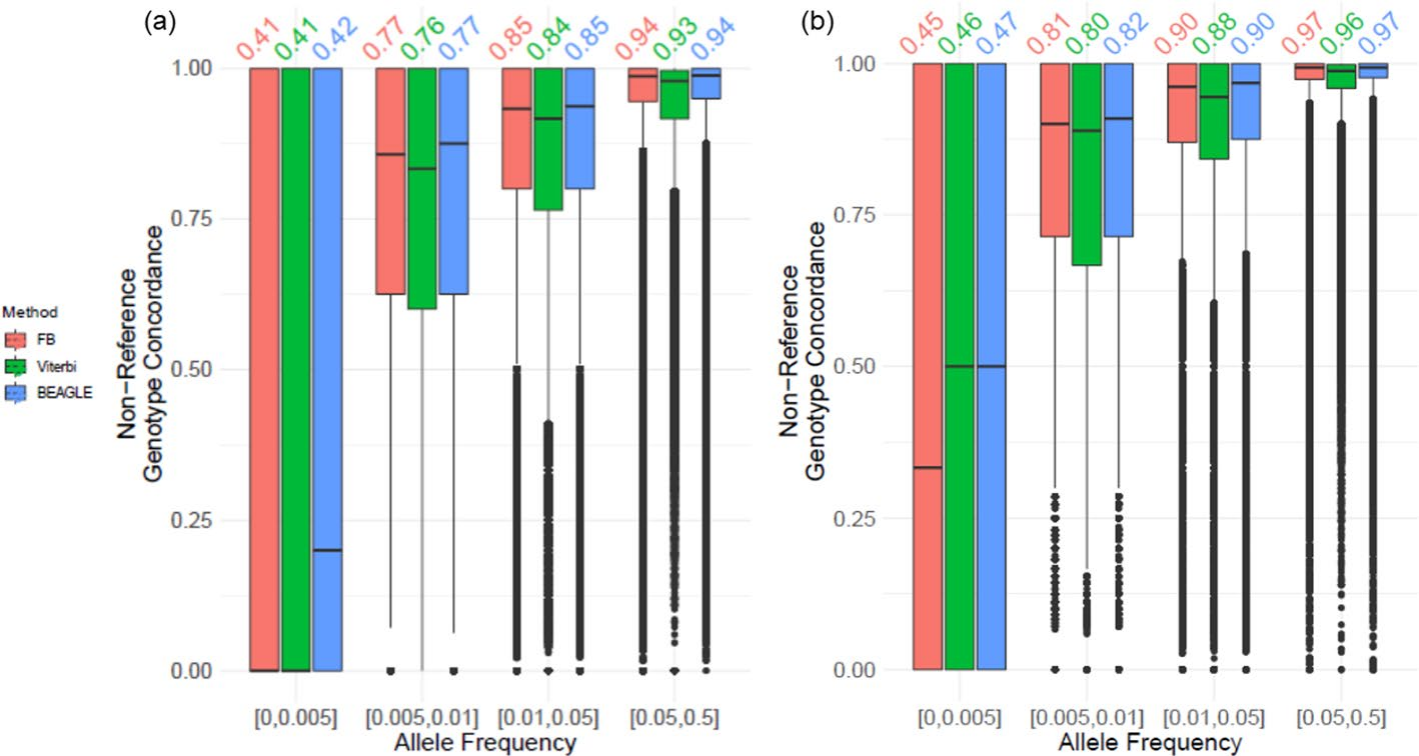
**a** Distribution of non-reference genotype concordance for the untyped variants on chromosome 19. The variants are stratified with respect to minor allele frequency (MAF) as shown on the x-axis. **b** Distribution of non-reference genotype concordance for the untyped variants on chromosome 20

In order to validate and compare the vicinity-based HMM parameter accuracy on a separate dataset, we tested the parameters for the variants on chromosomes 19 and 20. We extracted the typed variants on the Illumina Duo array platform on chromosomes 19 and 20. After this, we extracted 24,333 of 27,403 typed variants on chromosome 19 and 26,405 of the 28,319 typed variants on chromosome 20. The remaining variants (768,292 variants on chr19 and 742,370 on chr20) are used as untyped variants that are imputed by vicinity-based HMM and by BEAGLE. We classified the variants with respect to MAF by separating variants into 4 different MAF ranges: 1) $\mathrm{MAF}\in \left[0, 0.005\right]$ (Very rare), 2) $\mathrm{MAF}\in \left[0.005, 0.01\right]$ (Rare), 3) $\mathrm{MAF}\in \left[0.01, 0.05\right]$ (Uncommon), 4) $\mathrm{MAF}\in \left[0.05, 0.5\right]$ (Common). Before imputing untyped variants, the genotypes are phased using Eagle2 [49]. We use the parameters $\left({l}_{w},{N}_{e},{l}_{c2t},{n}_{tag}\right)=({\mathrm{0.5,10}}^{3},0.02, 1000)$ for validation of accuracy. Figure 8 shows the non-reference genotype concordance distribution for the variants on chromosome 19 (Fig. 8a) and chromosome 20 (Fig. 8b). The imputation of variants in the MAF range of common and uncommon are comparable with the baseline imputations of BEAGLE with less than 1% different in accuracy between baseline and vicinity-based HMM. The non-reference genotype concordance is less than 2 percent different for the rare variant categories. These results indicate that vicinity-based HMMs can potentially provide utility for uncommon and common variants (i.e., MAF > 1%).

## Discussion

We analyzed the feasibility of imputing variants using HMMs that are computed on locality of the target variants, i.e. untyped variants. There are several advantages of focusing exclusively to the locality of an untyped target. First, the computations can be parallelized and performed at a much smaller scale without the need of large number of untyped variants. Second, the evaluation of assessment of the vicinity-based HMM accuracy can provide biological insight into the haplotype structure and imputability estimates [43]. Third, the local models can be run in isolation from other parts of the genome. This way, the imputation algorithms can be re-designed for other tasks. For instance, recently developed privacy-aware imputation methods [47, 48] make extensive use of the vicinity-based models. Our results provide insight into the design of secure imputation algorithms so that they can appropriately select vicinity parameters to ensure sufficient resources are used while imputation is performed accurately. Also, our study provides evidence that HMM-based imputation methods can be designed with a pure vicinity-based approach. While we did not consider iterative approaches for estimation and tuning of parameters, the parameters can be optimized using, for example, Expectation–Maximization [63], specifically using Baum-Welch algorithm [55, 56].

Numerous optimizations can be introduced to decrease time and memory usage of vicinity-based HMMs. For example, forward and backward calculations can be re-ordered to streamline multiplications by using single-instruction-multiple-data (SIMD) operations. Also, we observed that the vicinity-windows substantially overlap while they are being computed for neighboring windows. Many of these do not have to be computed from scratch and can be re-used between neighboring windows. The imputation of untyped variants (especially the rare variants) that are very close to each other can be performed jointly as they are generally constrained to be on the same haplotype. Finally, some of the typed variants that are close to each other may not be providing extra information for imputation. These variants can be treated as a single unit while forward and backward variables are computed.

It should be noted that the default parameters do not provide the optimal performance that can be achieved using imputation HMMs that work on locality of untyped variants. For instance, we did not evaluate the impact of increasing ${l}_{w}$ while the maximum number of typed variants is kept constant. This would still constitute a vicinity-based HMM model since the maximum number of surrounding typed variants is constrained. In other words, this would keep the computational requirements constant but it would enable vicinity-based HMM to assess larger haplotype blocks. In addition, the locality windows can be implemented in different ways. For example, the typed variants can be filtered with respect to the smallest genetic distance, i.e., we can remove the typed variants that are close to each other and may only provide redundant information for imputation.

The main limitation of the vicinity-based HMM methods that are evaluated here is the lower accuracy for rare variants, especially for the variants with MAF lower than 1%. Our results show that the performance can be improved by extending the local windows to include more variants. This is reasonable since longer windows enable the resolution of the rare haplotypes more accurately than shorter windows. From a utility perspective, we observed that most of the downstream analyses, such as genomewide association studies (GWAS) impose thresholds on the well above 1% [64]. For instance, even high powered GWAS studies impose thresholds at 2–5% on the MAF of the variants to provide enough power for detecting phenotype-genotype associations [65]. Also, even the state-of-the-art HMM methods may not provide the imputation accuracy for low MAF variants that is necessary for the downstream analyses. Furthermore, these rare variants tend to be population-specific [66] and usage of population specific panels can enable more accurate performance. Thus, the vicinity-based HMMs can be used to impute variants for downstream tasks with MAF values that are utilizable for studies such as GWAS.

## Conclusions

Locality-based HMMs that are parametrized in this study can be used to parallelize and/or localize computations without the need to perform chromosome-wide (or very large scale) computations. We hypothesize that these methods can effectively be used for generating genotype imputations that are utilizable by downstream analyses such as GWAS. We believe one of the main uses of the vicinity-based HMMs is for using locality-based methods, which have been used in machine-learning-based and privacy-aware imputation models. The parametrizations can be used to guide the parameter selections in these methods.

## Methods

We present the computational details of the Viterbi and forward–backward estimation from the vicinity-based HMM.

### Description of the imputation HMMs within local window of untyped variants

LoHaMMer computes forward–backward (Fig. 1b) and Viterbi (Fig. 1c) estimates on the typed variants, i.e., keeps track of haplotype paths that are passing through only the typed variants. We assume that the genotypes are phased and the genotype matrix is denoted by ${G}_{i,j}^{\left(h\right)}$, which stands for the allele on parental copy $h$ for individual $i$ and the variant at index $j$. The parental copy has two values $h\in \left\{\mathrm{0,1}\right\}$, indicating the paternal and maternal haplotypes (or vice versa). ${G}_{i,[1,j]}^{\left(h\right)}$ indicates the sequence of alleles for *i*th individual for variants between 1 and *j*, i.e., ${G}_{i,[1,j]}^{\left(h\right)}=({G}_{i,1}^{\left(h\right)}, {G}_{i,2}^{\left(h\right)}, \dots ,{G}_{i,j}^{\left(h\right)})$. The alleles for each variant can have 2 values, ${G}_{i,j}^{\left(h\right)}\in \left\{\mathrm{0,1}\right\}$, denoting reference and alternate alleles. ${S}_{i,k}^{(h)}$ denotes the HMM state at the variant $k$ for *i*th individual. The states correspond to the indices of haplotypes in the phased reference genotype panel, i.e., ${S}_{i,k}^{(h)}\in [1,N]$. We denote the indices of the untyped variants with ${j}_{\varnothing }$, which is the set of variant indices (i.e., $j<V$) for which the genotypes are missing.

#### Variant subsampling

Given the maximum number of typed variants (or typed variants), ${\widehat{n}}_{tag}$, LoHaMMer first identifies all the variants in the current window, which is of length ${l}_{w}$. Given that ${n}_{tag}>{\widehat{n}}_{tag}$ is the total number of variants, LoHaMMer takes every ${\left(\frac{{\widehat{n}}_{tag}}{{n}_{tag}}\right)}^{th}$ variant to select ${\widehat{n}}_{tag}$ in the window. If ${n}_{tag}$ is smaller than ${\widehat{n}}_{tag}$, all the typed variants are used for imputation. To simplify the presentation, we assume that the variant indexing is based on the subsampled variant list.

#### Computation of genetic distance at the typed and untyped variants $({\mathbf{R}}_{\mathbf{k}})$

The genetic distance in the unit of centimorgan is a probabilistic measure of how likely two variants are shared in same haplotype block in meiosis. We use genetic distance to define the window length parameter (${l}_{w}$) around the untyped variants. Numerous previous studies have generated genetic maps as references using estimated recombination patterns on the human genome. For each chromosome, these maps tabulate an estimate of the cumulative genetic distances from the beginning of the chromosome to a set of dense markers that are used in estimation of genetic distances. The markers used in estimation of genetic distances do not necessarily overlap with the typed variants that are used in imputation. We therefore need to interpolate the genetic distance of each typed variant. Given a ${k}^{th}$ typed (or untyped) variant whose genomic coordinate is denoted by ${pos}_{k}$ base pairs, we use a lookup table to identify the closest two genetic distance markers with genomic coordinates $l$ and $m$, such that $l\le {pos}_{k}\le m$. As $m$ is greater than $l$, the cumulative genetic distance is larger or equal to the distance for $l$, i.e., $\Delta \left(m\right)\ge\Delta (l)$, where $\Delta (m)$ indicates the cumulative genetic distance of the marker located at genomic coordinate $m$. We estimate the cumulative genetic distance of the typed variant $k$ using a linear interpolation of the distances at $l$ and $m$ weighted by genomic distance:

11 $${R}_{k}=\Delta \left(l\right)+\left(\frac{\left(\Delta \left(m\right)-\Delta \left(l\right)\right)}{(m-l)}\times \left({pos}_{k}-l\right)\right)$$

This is performed separately for each chromosome. After the assignment of genetic distances to all typed and untyped variants, the differences between the distances, e.g., $\left|{R}_{k}-{R}_{n}\right|$, is used in parameter benchmarks. We use prebuilt estimates of genetic distances from the IMPUTE2 web site (https://mathgen.stats.ox.ac.uk/impute/1000GP_Phase3.html).

#### Marginal probability estimation by forward–backward algorithm

The forward–backward algorithm relies on computation of forward and backward variables. Given individual $i$ and haplotype $h$, the forward probability is formulated as

12 $$P\left( {S_{{i,j}}^{{\left( h \right)}} = a,~G_{{i,\left[ {1,j} \right]}}^{{\left( h \right)}} ~} \right) = \mathop \sum \limits_{{\begin{array}{*{20}c} {\forall S_{{i,\left[ {1,j} \right]}} } \\ {S_{{i,j}} = a} \\ \end{array} }} \left( {\mathop \prod \limits_{{1 \le k \le j}} \left( {\underbrace {{\tau _{k} \left( {S_{{i,k - 1}} \to S_{{i,k}} } \right)}}_{{\mathop {Transition~from~}\limits_{{S_{{i,k - 1}} ~to~S_{{i,k}} }} }} \times \underbrace {{e_{k} \left( {G_{{i,k}}^{{\left( h \right)}} ,S_{{i,k}} } \right)}}_{{\mathop {Emission~of~G_{{i,k}}^{{\left( h \right)}} }\limits_{{from~the~state\,S_{{i,k}} }} }}} \right)} \right)$$

where $P\left({S}_{i,j}^{(h)}=a, {G}_{i,[1,j]}^{(h)}\right)$ denotes the forward variable, which is the total probability of all state sequences and the emissions from the state sequences ${S}_{i,\left[1,j\right]}$ that emit the allele sequence ${G}_{i,[1,j]}^{(h)}$ with the constraint that the last state at variant $j$ is $a$, i.e., ${S}_{i,j}^{(h)}=a$. The forward variable matrix can be computed recursively [55, 56] for all variant positions and all states using

13 $$P\left( {S_{{i,j}}^{{\left( h \right)}} = a,~G_{{i,\left[ {1,j} \right]}}^{{\left( h \right)}} } \right) = \mathop \sum \limits_{{1 \le b \le N}} \left( {\underbrace {{P\left( {S_{{i,j - 1}}^{{\left( h \right)}} = b,~G_{{i,\left[ {1,j - 1} \right]}}^{{\left( h \right)}}} \right)}}_{{\mathop {Forward~variable}\limits_{{at~\left( {j - 1} \right)}} }} \times \tau _{b} \left( {b \to a} \right) \times e_{j} \left( {G_{{i,j}}^{{\left( h \right)}} ,a} \right)} \right)$$

where the forward variable at variant $j$ is computed using the forward variable at position $(j-1)$. The boundary condition is defined at the first nucleotide:

14 $$\forall a\in \left[1,N\right], P\left({S}_{i,0}^{(h)}=a , {G}_{i,\left[\mathrm{1,0}\right]}^{\left(h\right)}\right)=\frac{1}{N}$$

which indicates that the state at the first variant is uniformly distributed among all states, i.e. there is no preference between haplotypes that initiate the HMM. This boundary condition is sometimes described by introducing a special state named the “start state”.

The backward probability is formulated as

15 $$P\left( {G_{{i,\left[ {j + 1,V} \right]}}^{{\left( h \right)}} |S_{{i,j}}^{{\left( h \right)}} = a} \right) = \mathop \sum \limits_{{\begin{array}{*{20}c} {\forall S_{{i,\left[ {j,V} \right]}} } \\ {S_{{i,j}} = a} \\ \end{array} }} \left( {\mathop \prod \limits_{{j \le k \le V}} \left( {\underbrace {{\tau _{k} \left( {S_{{i,k}} \to S_{{i,k + 1}} } \right)}}_{{\mathop {Transition~from~}\limits_{{S_{{i,k}} ~to~S_{{i,k + 1}} }} }} \times \underbrace {{e_{k} \left( {G_{{i,k + 1}}^{{\left( h \right)}} ,S_{{i,k + 1}} } \right)}}_{{\mathop {Emission~of~G_{{i,k + 1}}^{{\left( h \right)}} }\limits_{{from~the~state\,S_{{i,k + 1}} }} }}} \right)} \right)$$

In (15), $P\left({G}_{i,[j+1,V]}^{\left(h\right)} | {S}_{i,j}^{(h)}=a\right)$ denotes the backward variable for *i*th individual’s haplotype $h$, and the total probability over all the state sequences, ${S}_{i,\left[j,V\right]}^{(h)}$, that emit the allele subsequence ${G}_{i,[j+1, V]}^{\left(h\right)}$ with the constraint that the first state at variant $j$ is $a$, i.e., ${S}_{i,j}^{(h)}=a$. The backward variable can be computed using a recursion relationship [55, 56] using following:

16 $$P\left( {G_{{i,\left[ {j + 1,V} \right]}}^{{\left( h \right)}} |S_{{i,j}}^{{\left( h \right)}} = a} \right) = \mathop \sum \limits_{{1 \le b \le N}} \left( {\underbrace {{P\left( {G_{{i,\left[ {j + 2,V} \right]}}^{{\left( h \right)}} |S_{{i,j + 1}}^{{\left( h \right)}} = b} \right)}}_{{\mathop {Backward~variable~}\limits_{{at~\left( {j + 1} \right)}} }} \times \tau _{j} \left( {a \to b} \right) \times e_{j} \left( {G_{{i,j + 1}}^{{\left( h \right)}} ,b} \right)} \right)$$

The boundary condition for backward variable is set for the ends of windows:

17 $$\forall a\in \left[1,N\right], P\left({G}_{i,\left[V+1, V\right]}^{\left(h\right)} | {S}_{i,\left(V+1\right)}^{(h)}=a \right)=\frac{1}{N}$$

which indicates that the haplotypes are uniformly distributed at the end of the allele sequence. The emission and transition probabilities are accordingly set to uniform at the boundaries of forward and backward variables.

#### Computation of the allele and genotype probabilities for untyped variants

The forward and backward variables are used for inferring the probability of observing alleles 0 and 1 at the untyped variants. To estimate the allele probabilities of an untyped variant at index $j\in {j}_{\varnothing }$, LoHaMMer identifies the two consecutive typed variants that are closest to the variant $j$. Using the nearest typed variant, LoHaMMer uses an approach similar to BEAGLE to estimate the path that passes along the untyped variant as

18 $$\forall j\in {j}_{\varnothing }, P\left({G}_{i,j}^{\left(h\right)}=t\right)=\sum_{\begin{array}{c}1\le a\le N\\ {H}_{a,j}=t\end{array}}\left(P\left({S}_{i,k}^{\left(h\right)}=a, {G}_{i,\left[1,k\right]}^{\left(h\right)}\right)\times P\left({G}_{i,\left[k+1,V\right]}^{\left(h\right)} | {S}_{i,k}^{\left(h\right)}=a\right)\right), t\in \{\mathrm{0,1}\}$$

where ${j}_{\varnothing }$ indicates the untyped variant indices in the genotype matrix, and $k$ is the variant index such that variants at $k$ and $\left(k+1\right)$ are the closest typed variants to the untyped variant $j$. The allelic probabilities from the parental copies are normalized and combined to generate a final genotype probability for the 3 possible genotypes, i.e., combinations of the alleles assigned to the two haplotypes of an individual. The genotype probabilities are computed as

19 $$P\left({G}_{i,j}=0\right)=P^{\prime}\left({G}_{i,j}^{\left(0\right)}=0\right)\times P^{\prime}\left({G}_{i,j}^{(1)}=0\right)$$

20 $$P\left({G}_{i,j}=1\right)=P^{\prime}\left({G}_{i,j}^{(0)}=0\right)\times P^{\prime}\left({G}_{i,j}^{(1)}=1\right)+P^{\prime}\left({G}_{i,j}^{\left(0\right)}=1\right)\times P^{\prime}\left({G}_{i,j}^{(1)}=0\right)$$

21 $$P\left({G}_{i,j}=2\right)=P^{\prime}\left({G}_{i,j}^{\left(0\right)}=1\right)\times P^{\prime}\left({G}_{i,j}^{(1)}=1\right)$$

where $P^{\prime}\left({G}_{i,j}^{\left(h\right)}=t\right)$ ($t\in \left\{\mathrm{0,1}\right\}, h\in \{\mathrm{0,1}\}$) denotes the normalized allelic probability for haplotype $h$ and allele $t$. $P^{\prime}\left({G}_{i,j}^{\left(h\right)}=t\right)$ is computed by normalizing with respect to the total allelic probability for the variant so that the range is in $[\mathrm{0,1}]$. Specifically, we use

22 $$P^{\prime}\left({G}_{i,j}^{\left(h\right)}=t\right)=\frac{P^{\prime}\left({G}_{i,j}^{(h)}=t\right)}{P^{\prime}\left({G}_{i,j}^{(h)}=t\right)+P^{\prime}\left({G}_{i,j}^{(h)}=1-t\right)}$$

where the normalization is performed over the two possible allelic probabilities for the parental copy $h$ for allele $t$.

#### Maximum-likelihood haplotype path estimation by Viterbi algorithm

Similar to the forward matrices, Viterbi method keeps track of the maximum scoring matrix at each typed variant for every possible haplotype state:

23 $$P^{\wedge} \left( {\overset{\lower0.5em\hbox{$\smash{\scriptscriptstyle\smile}$}}{S}_{{i,j}}^{{\left( h \right)}} = a,~G_{{i,\left[ {1,j} \right]}}^{{\left( h \right)}} } \right) = \mathop {\max }\limits_{{\begin{array}{*{20}c} {\forall S_{{i,\left[ {1,j} \right]}} } \\ {S_{{i,j}} = a} \\ \end{array} }} \left( {\mathop \prod \limits_{{1 \le k \le j}} \left( {\underbrace {{\tau _{k} \left( {S_{{i,k - 1}} \to S_{{i,k}} } \right)}}_{{\mathop {Transition~from~}\limits_{{S_{{i,k - 1}} ~to~S_{{i,k}} }} }} \times \underbrace {{e_{k} \left( {G_{{i,k}}^{{\left( h \right)}} ,S_{{i,k}} } \right)}}_{{\mathop {Emission~of~G_{{i,k}}^{{\left( h \right)}} }\limits_{{from~the~state\,S_{{i,k}} }} }}} \right)} \right)$$

where $$P^{ \wedge }\left({\overset{\lower0.5em\hbox{$\smash{\scriptscriptstyle\smile}$}}{S}}_{i,j}^{(h)}=a, {G}_{i,[1,j]}^{(h)}\right)$$ indicates the probability of the typed allele sequence $${G}_{i,[1,j]}^{(h)}$$ emitted by the most likely state sequence $${\check{S}}_{i,[1,j]}^{(h)}$$ with additional constraint of $${\check{S}}_{i,j}^{(h)}=a$$. This path is the most likely path that LoHaMMer uses to infer the most likely haplotype mosaic that emits the typed allele sequence. (23) is exactly same as forward variable in (12) except that the leftmost summation in (12) is replaced with a maximum operator. Therefore, $$P^{ \wedge }\left({\overset{\lower0.5em\hbox{$\smash{\scriptscriptstyle\smile}$}}{S}}_{i,j}^{(h)}=a, {G}_{i,[1,j]}^{(h)}\right)$$ can be computed using a similar recursion by replacing the summation with a maximum operator in (13):

24 $$P^{\wedge} \left( {\check{S}_{{i,j}}^{{\left( h \right)}} = a,~G_{{i,\left[ {1,j} \right]}}^{{\left( h \right)}} } \right) = \mathop {\max }\limits_{{1 \le b \le N}} \left( {\underbrace {{P^{\wedge} \left( {\check{S}_{{i,j - 1}}^{{\left( h \right)}} = b,~G_{{i,\left[ {1,j - 1} \right]}}^{{\left( h \right)}} } \right)}}_{{\mathop {Viterbi~variable~}\limits_{{at~\left( {j - 1} \right)}} }} \times \tau _{b} \left( {b \to a} \right) \times e_{j} \left( {G_{{i,j}}^{{\left( h \right)}} ,a} \right)} \right)$$

LoHaMMer computes the Viterbi matrix using this recursion relationship for every typed variant from left to right for all the haplotypes with the boundary condition:

25 $$\forall a\in \left[1,N\right], P^{ \wedge }\left({\check{S}}_{i,0}^{(h)}=a,{ G}_{i,[\mathrm{1,0}]}^{\left(h\right)}\right)=\frac{1}{N}$$

As for the forward and backward matrices, the Viterbi matrix is computed over the typed variants.

After computing the Viterbi matrix, LoHaMMer traces back the Viterbi matrix to identify the optimal state sequence i.e., the optimal set of haplotypes that emits the full allelic sequence:

26 $${\overset{\lower0.5em\hbox{$\smash{\scriptscriptstyle\smile}$}}{S}}_{i,\left[1,V\right]}^{\left(h\right)}=\underset{{S}_{\left[1,V\right]}}{\mathrm{argmax}}\left\{P\left({S}_{\left[1,V\right]}, {G}_{i,\left[1,V\right]}^{\left(h\right)}\right)\right\}$$

After the optimal state is assigned, LoHaMMer assigns the alleles to the untyped variants similar to the forward–backward algorithm. For the untyped variant at index $j$, LoHaMMer identifies the closest typed variant and assigns the allele based on the maximum-likelihood state on the typed variant:

27 $$\forall j\in {j}_{\varnothing }, {\overset{\lower0.5em\hbox{$\smash{\scriptscriptstyle\smile}$}}{G}}_{i,j}^{\left(h\right)}={H}_{t,k} ;t= {\overset{\lower0.5em\hbox{$\smash{\scriptscriptstyle\smile}$}}{S}}_{i,j}^{\left(h\right)}$$

where $k$ is the typed variant that is closest to the untyped variant at $j$.

#### Haplotype clustering in blocks of variants

The recursion relationships for Viterbi and forward–backward variables indicate that it is necessary to perform a summation (or a maximum operation) over all the haplotypes in the reference panel, for every typed variant. This computation can become quickly intractable as the number of haplotypes ($N$) increases. Similar to the previous methods, LoHaMMer clusters the haplotypes, computes each forward, backward, and Viterbi arrays over the clusters of reference haplotypes to minimize the number of redundant operations. The clustering increases the efficiency substantially because (1) the number of unique haplotypes over short stretches increase much slower compared to the number of haplotypes, (2) the transition probabilities between states depends only on the self-transition and recombinations. These optimizations are extensively described in previous methods. We briefly describe the usage of clustering for computation of Viterbi arrays. LoHaMMer selects a number of variants that will be used to cluster the reference haplotypes, by default the block length is selected to be 10 variants. Given a local window, LoHaMMer divides the window into blocks of 10 variants. Next the reference haplotypes on each block are clustered such that each cluster corresponds to a unique sequence of 10 alleles, corresponding to 10 variants in the block. Next, for each cluster, the Viterbi variable is computed as the maximum of the Viterbi variable over the haplotypes in the cluster. Since the clusters share the allelic sequence exactly, Viterbi variables for the clusters are computed at the cluster-level using the recursion relatonships over the 10 variants in the block. After cluster-level Viterbi variables are computed for each cluster, LoHaMMer assigns the Viterbi variable to each haplotype from their corresponding cluster-level Viterbi variables.

#### Numerical stability

The transition and emission probabilities are smaller than 1 and they are multiplied with each other over all transitions and emissions. Thus, the Viterbi variable and forward–backward variables may overrun or underrun the numerical precision. To get around these numerical stability isssues, LoHaMMer can perform the computations in the logarithmic domain or it scales the Viterbi and forward–backward variables by a scaling factor. For the logarithmic domain computations, LoHaMMer keeps every value as logarithms. In logarithmic domain, a multiplication is converted to a summation and this is convenient since the overflow is virtually impossible. However, we observed that the approximate summation in logarithmic domain requires numerous slow operations (summation in log domain requires exponentiation) and increases time requirements. Therefore, LoHaMMer uses a linear scaling value by default. For this, LoHaMMer multiplies every array value by a constant scaling factor. We observed by trial-and-error that scaling factor of $\mathrm{exp}(0.2)$ enables minimal number of underflow or overflow issues. LoHaMMer keeps track of any overflow and underflow at each computation step. If an array value becomes too high or too low, the values are re-scaled to ensure numerical stability.

### Computation of accuracy metrics

#### Non-reference genotype concordance

The genotype concordance is computed as the overlap between the genotypes that are known to be non-reference. More formally, this is formulated as

28 $${\kappa }_{j}^{(NR)}=\frac{|\left\{i \right|{G}_{i,j}^{Known}>0\} \cap {G}_{\cdot ,j}|}{\left|\left\{i \right|{G}_{i,j}^{Known}>0\}\right|}$$

where ${\kappa }_{j}^{(NR)}$ denotes the non-reference concordance between the known non-reference genotypes of variant $j$ and the imputed variants over all individuals.

### Data sources

The 1000 genomes project genotypes are downloaded from NCBI ftp data portal. The Illumina Duo v3 variants are extracted from the array’s documentation available at: https://zenodo.org/record/5482126#.YTcAEM9On3g. The variants in The 1000 Genomes Project that overlap with the variants on the array’s typed variants are used as the typed variants.

## Supplementary Information

**Additional file 1.** This additional file contains the supplementary Text and Figures with extended discussion and accuracy results.

## Data Availability

The 1000 genomes project genotypes are downloaded from NCBI ftp data portal at http://ftp.1000genomes.ebi.ac.uk/vol1/ftp/release/20130502/. The Illumina Duo v3 variants are extracted from the array’s documentation available at: https://zenodo.org/record/5482126#.YTcAEM9On3g. The variants in The 1000 Genomes Project that overlap with the variants on the array’s typed variants are used as the typed variants. The source code for vicinity-based imputation, data processing scripts, and intermediate data files can be reached from https://github.com/harmancilab/LoHaMMer. The genetic maps on hg19 human chromosomes can publicly be accessed from https://mathgen.stats.ox.ac.uk/impute/1000GP_Phase3.html.
