# Supplementary material for: Evaluation of vicinity-based hidden Markov models for genotype imputation
Source: BMC Bioinformatics. 2022 Aug 29;23:356. doi: 10.1186/s12859-022-04896-4 (PMC9422108; doi:10.1186/s12859-022-04896-4)
Supplement: Supplementary file 1 — Additional file 1. This additional file contains the supplementary Text and Figures with extended discussion and accuracy results. [file 12859_2022_4896_MOESM1_ESM.pdf]

Supplementary Information for “Evaluation of Vicinity-based Hidden Markov Models for Genotype Imputation”

Su Wang[1], Miran Kim[2], Xiaoqian Jiang[3], Arif Ozgun Harmanci[1,*]

1 Center for Precision Health, School of Biomedical Informatics, University of Texas Health Science Center, Houston, TX, 77030, United States of America.

2 Department of Computer Science and Engineering and Graduate School of Artificial Intelligence, Ulsan National Institute of Science and Technology, Ulsan, 44919, Republic of Korea.

3 Center for Secure Artificial intelligence For hEalthcare (SAFE), School of Biomedical Informatics, University of Texas Health Science Center, Houston, TX, 77030, United States of America.

*: Corresponding author, email: arif.o.harmanci@uth.tmc.edu

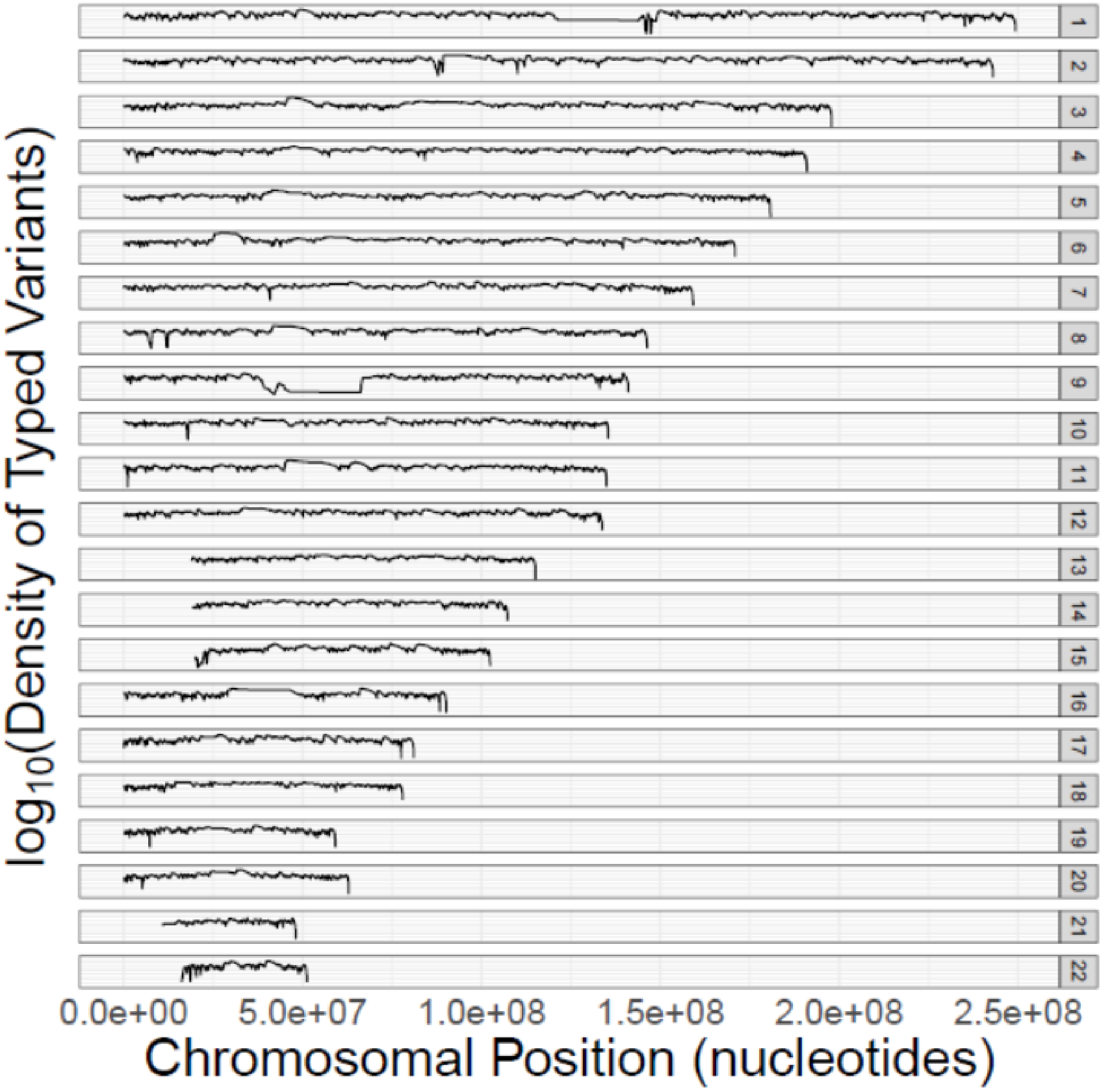

Supplementary Figure 1: The typed variant density for 22 chromosomes. Each chromosome is shown on the rows. X-axis shows the chromosomal position and y-axis shows the logarithm of the number of typed variants in the range.

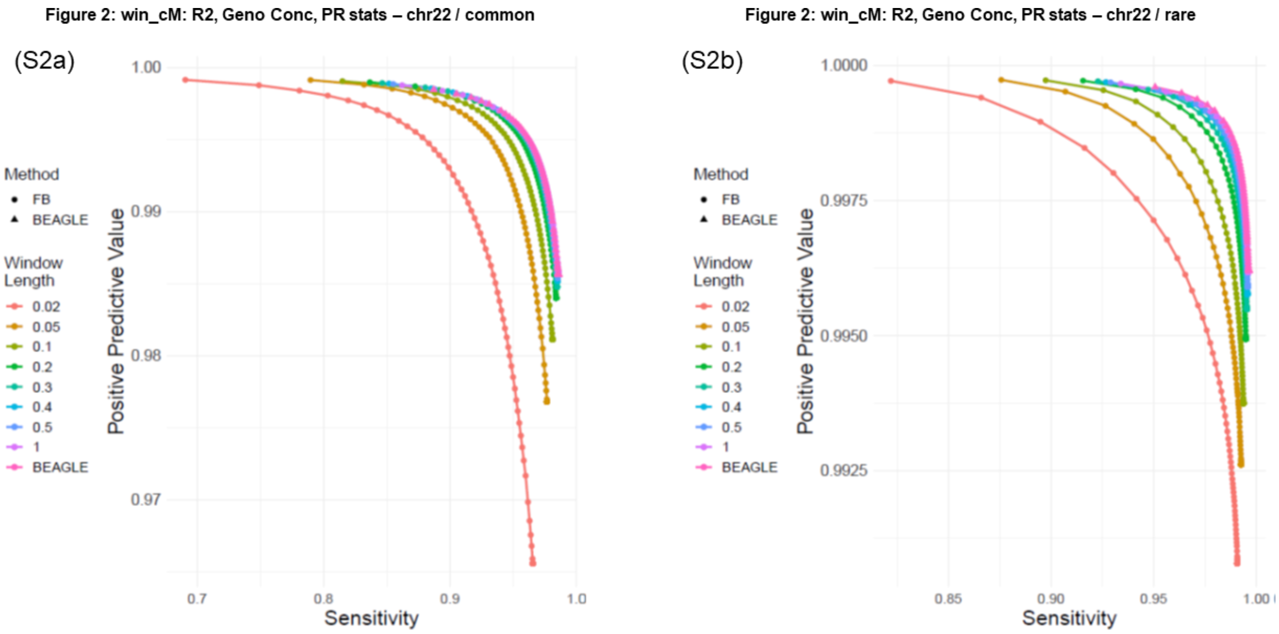

Supplementary Figure 2: The genotype precision-recall curves for common variants (S2a) and non-common variants (S2b) with changing window length parameter.

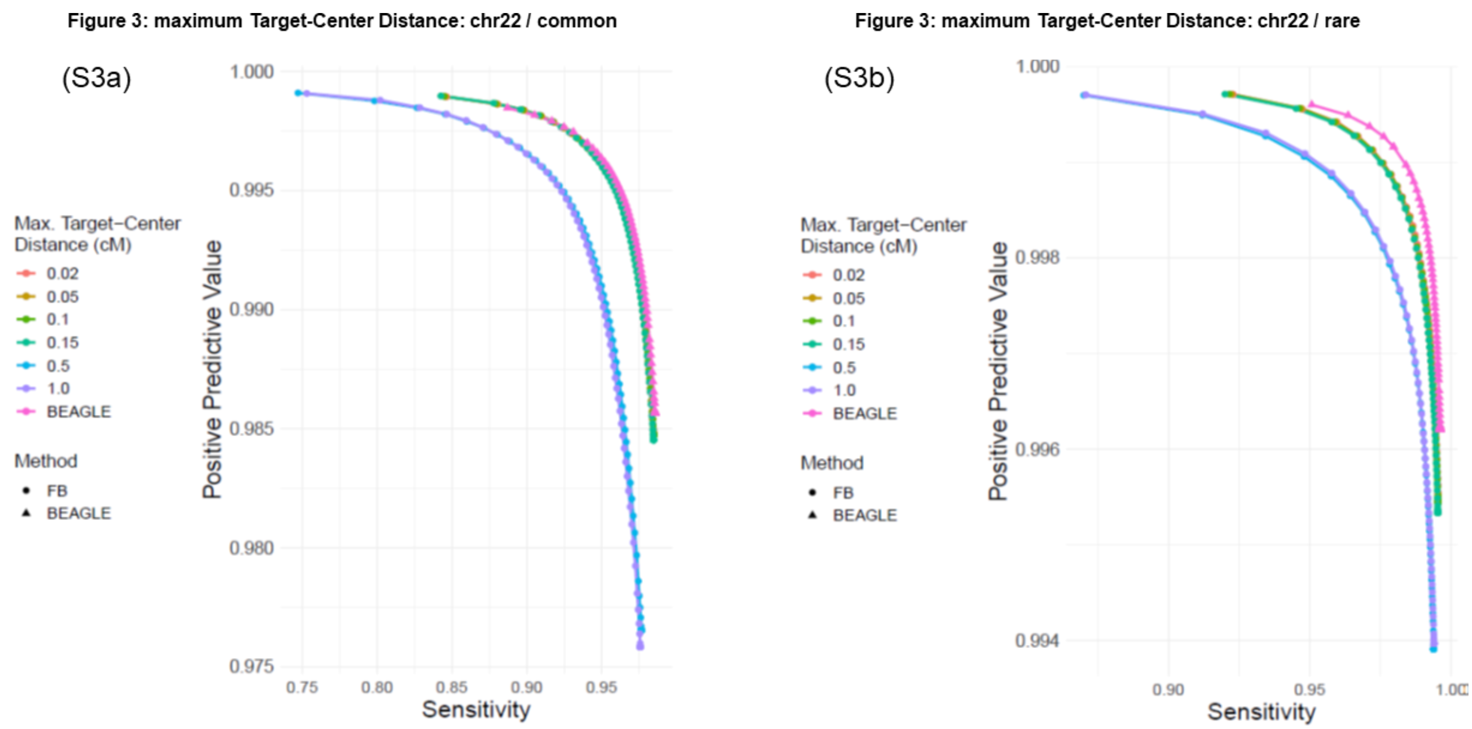

Supplementary Figure 3: The genotype precision-recall curves for common variants (S3a) and for uncommon variants (S3b) with changing maximum target-center distance.

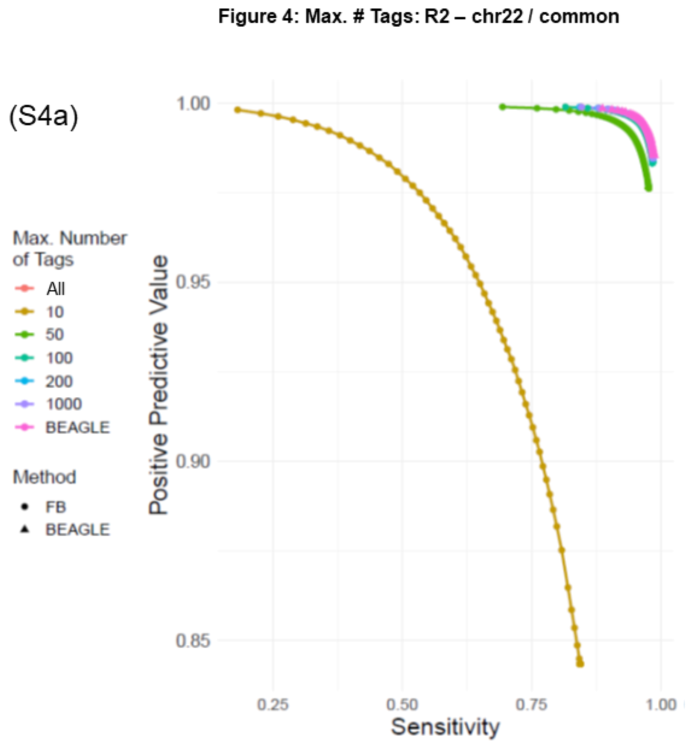

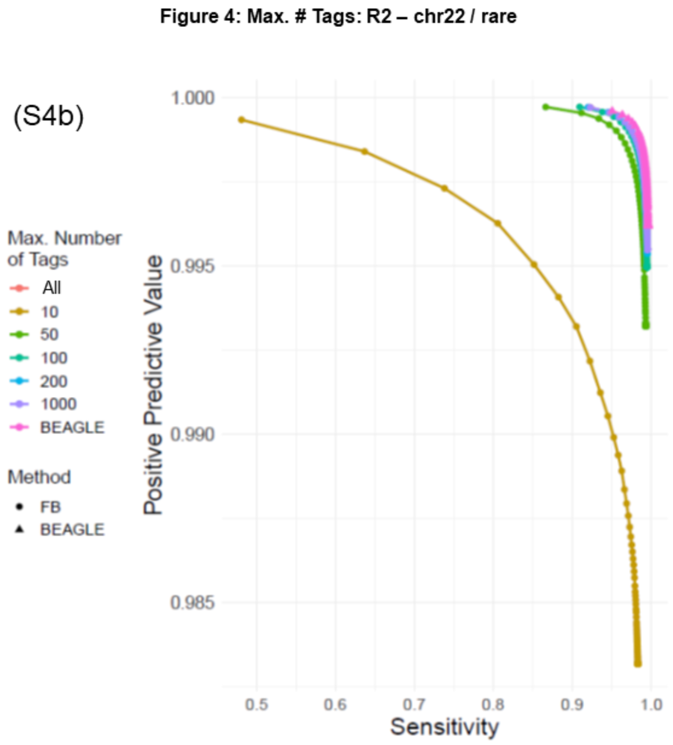

Supplementary Figure 4: The genotype precision-recall curves for common variants (S4a) and un-common variants (S4b) with changing maximum typed variants in window.
